# Molecular diagnostic assays based on *cpn60* UT sequences reveal the geographic distribution of subgroup 16SrXIII-(A/I)I phytoplasma in Mexico

**DOI:** 10.1038/s41598-017-00895-1

**Published:** 2017-04-19

**Authors:** Edel Pérez-López, Douglas Rodríguez-Martínez, Chrystel Y. Olivier, Mauricio Luna-Rodríguez, Tim J. Dumonceaux

**Affiliations:** 1 Instituto de Biotecnología y Ecología Aplicada (INBIOTECA), Universidad Veracruzana, Xalapa, Veracruz Mexico; 2 Departamento de Investigación Aplicada, Driscoll’s, Zapopan, Jalisco Mexico; 3 Agriculture and Agri-Food Canada, London Research and Development Centre, London, Ontario Canada; 4 Laboratorio de Genética e Interacciones Planta Microorganismos, Facultad de Ciencias Agrícolas, Universidad Veracruzana, Xalapa, Veracruz Mexico; 5 Agriculture and Agri-Food Canada, Saskatoon Research and Development Centre, Saskatoon, Saskatchewan Canada; 6 Department of Entomology and Plant Pathology, Auburn University, Auburn, AL 36849 USA

## Abstract

Geographically diverse samples from strawberry exhibiting symptoms of Strawberry Green Petal (SbGP), periwinkle plants with virescence, and blackberry, blueberry, and raspberry plants displaying yellowing and inedible fruits, were assayed for the presence of phytoplasma DNA. PCR targeting the 16S rRNA-encoding gene and chaperonin-60 (*cpn*60) showed that the plants were infected with phytoplasma subgroup16SrXIII-(A/I)I (SbGP/MPV). To examine the geographic distribution of this pathogen in Mexico, we designed an array of *cpn60*-targeted molecular diagnostic assays for SbGP/MPV phytoplasma. A fluorescent microsphere hybridization assay was designed that was capable of detecting SbGP/MPV phytoplasma in infected plant tissues, successfully differentiating it from other known phytoplasma *cpn*60 UT sequences, while identifying a double infection with SbGP/MPV and aster yellows (16SrI) phytoplasma. Two quantitative assays, quantitative real-time PCR (qRT-PCR) and droplet digital PCR (ddPCR), gave similar results in infected samples. Finally, a loop-mediated isothermal amplification (LAMP) assay provided rapid detection of SbGP/MPV phytoplasma DNA. Application of these assays revealed that SbGP/MPV phytoplasma is widely distributed in Central Mexico, with positive samples identified from eleven localities within three states separated by hundreds of kilometres. These results also provide tools for determining the presence and geographic distribution of this pathogen in plant and insect samples in other localities.

## Introduction

Phytoplasmas (‘*Candidatus* Phytoplasma’ spp.) are wall-less bacteria that were first described as mycoplasma-like organisms^1^ with a small, A-T rich and distinctive genome, that are taxonomically classified as Mollicutes^2, 3^. Phytoplasmas are insect-vectored plant pathogens that infect a very wide variety of plants, including most crop species, causing developmental alterations leading to leaf-like floral structures and aborted seed production^4–6^. Detection of phytoplasma infection in plant and insect tissues, along with proper classification and identification, is therefore critical for disease surveillance and control^2^. However, phytoplasmas are unculturable microorganisms and the detection and classification criteria are based on the use of molecular approaches^7^. Phytoplasma detection has relied on PCR amplification of targets within and surrounding the 16 S rRNA-encoding gene^8^. Identification and classification has typically used restriction fragment length polymorphism (RFLP) analysis of this locus^9^, resulting in the identification of over thirty 16Sr groups described as16SrI – 16SrXXXIII^10^. However, other genes have been used as additional markers, including the *groEL* or *cpn60* gene^11, 12^. The *cpn60* universal target (*cpn60* UT), a sequence of approximately 550 bp, is located within the Cpn60-encoding gene^13^. This sequence has been identified as a molecular barcode for the domain Bacteria^14^ and is used as a taxonomic marker to characterize microbial communities^15, 16^. Furthermore, *cpn60* UT has been shown to be a suitable target for the development of highly discriminatory molecular diagnostic assays for various organisms, including phytoplasma^12, 17^. The *cpn60* UT has also been identified as a marker to identify and classify phytoplasmas based on the *in silico* RFLP analysis of these sequences^18^.

Strawberry green petal (SbGP) disease affects strawberry plants (*Fragaria x ananassa*) and is associated with phytoplasmas^19^. This disease, which was first detected in 1959 in Central Europe^20^ has since been reported in Canada^21^, the Czech Republic^19^ and Italy^22^, and is typically associated with phytoplasmas of the Aster Yellows (‘*Ca*. Phytoplasma asteris’, 16SrI) group. The hallmarks of SbGP include the flower petals changing from white to green in color, along with fruits showing green structures that give the appearance of a large green flower. Another characteristic symptom of the disease is the presence of red leaves and the formation of leaves in the fruit, which renders the fruit inedible and not viable for commercial sale. In Australia and the USA, diseases associated with phytoplasma affecting strawberry plants are associated mainly with members of the Aster Yellows group^23, 24^. In Latin America, SbGP disease was first reported in Argentina and was found to be caused by phytoplasmas of the 16SrVII group^25^. Phytoplasmas from group 16SrXIII have also been associated with strawberry diseases, specifically strains included in subgroups 16SrXIII-B and 16SrXIII-F^24, 26^. The group 16SrXIII or Mexican periwinkle virescence was first identified in *Catharanthus roseus* from Mexico^27^ and represents a new ‘*Ca*. Phytoplasma’ species, ‘*Ca*. Phytoplasma hispanicum’^28^. Other plants, such as potato, have also been identified as hosts, but members of this phytoplasma group have not been described outside of the Americas^29^.

We previously determined that Strawberry green petal (SbGP) disease affecting strawberry plants and Mexican periwinkle virescence disease (MPV) affecting periwinkle plants (*Catharanthus roseus*) in Mexico is associated with the heterogeneous 16SrXIII-(A/I)I phytoplasma^30^. In this work, we compared the performance of the previously reported PCR targeting phytoplasma *cpn60* ^12^ to nested PCR targeting the F2nR2 region^31^, an accepted standard for the detection of phytoplasma infections. To determine the presence and geographic distribution of this pathogen in Mexican production fields, we developed and applied *cpn60* UT-targeted molecular diagnostic assays to 86 samples of symptomatic strawberry, raspberry, blueberry, blackberry, and periwinkle plants sampled in 11 localities within the states of San Luis Potosi, Jalisco, and Michoacan, Mexico. The results reveal the minimum extent of the geographic distribution of this pathogen in Mexico and provide a set of tools for determining the prevalence and distribution of the pathogen in other geographic locations.

## Results

### Plants affected by 16SrXIII-(A/I)I phytoplasma are found in three Mexican states

In a previous study the presence of the SbGP/MPV phytoplasma [16SrXIII-(A/I)I] was demonstrated in samples of periwinkle from San Luis Potosi and of strawberry from Michoacan, Mexico^30^. This phytoplasma contains two non-identical copies of the 16S rRNA-encoding locus^30^. To confirm that the samples from all geographic areas in the present study represented the same strain, F2nR2 sequences were analyzed in samples S07-P-JC and S10-L-JC, which were randomly selected from the samples collected in the state Jalisco. The sequencing showed that clones representing both subgroups 16SrXIII-A and 16SrXIII-I^30^ were found in each sample (Supplementary Fig. S1). Furthermore, the *cpn60* UT sequences determined from 11 strawberry (KY061173 to KY061183), 1 blueberry (KY061168), 4 raspberry (KY061169 to KY061172), and 2 blackberry (KY061184, KY061185) samples were identical to the SbGP/MPV phytoplasma *cpn60* UT reported previously^30^ (e.g. GenBank accession no. KU896201). These results indicate that the phytoplasma affecting the samples was the 16SrXIII-(A/I)I phytoplasma previously identified. The F2nR2 sequences obtained for both samples were deposited to GenBank under the accession numbers: KY061162 and KY061163 for S07-P-JC-clone1, S07-P-JC-clone5, respectively, and KY061164 and KY061165 for S10-L-JC-clone3 and S10-L-JC-clone6, respectively.

### Performance of phytoplasma-targeted conventional PCR

**Figure 1:**
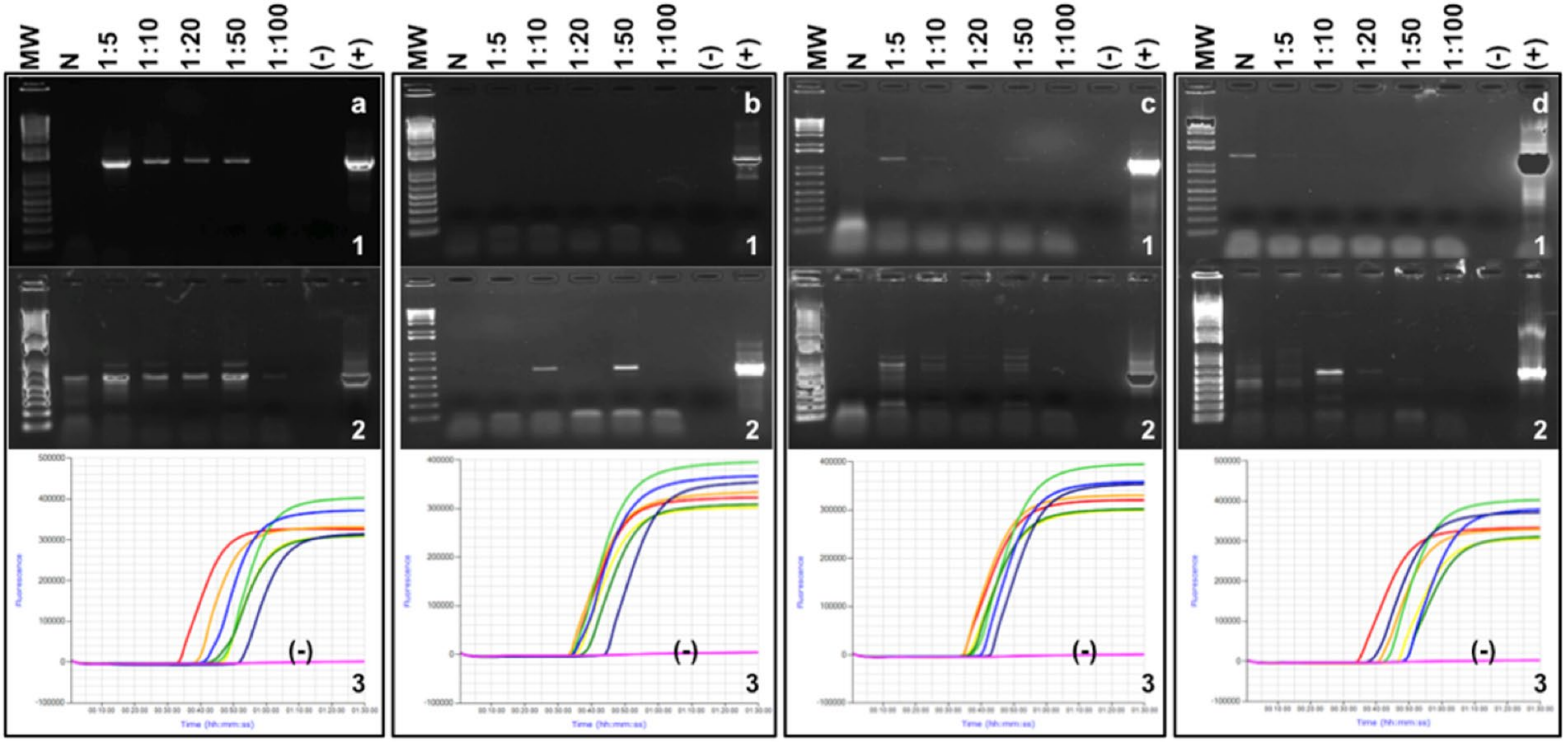
F2nR2-based PCR (**1**) and *cpn60*-based PCR (**2**) and LAMP amplification curves (**3**) applied to serial dilutions of infected berry plant DNA analyzed in this study. (**a)** Sample S05-L-MB (strawberry); (**b)** Bl01-L-JA (blueberry); (**c**) R05-L-MC (raspberry); (**d)** Bk02-L-JD (blackberry). No positive controls were included in the LAMP assay (**3**), and negative (−) in all cases refers to the “no template” control.

DNA extracts were analyzed using pan-phytoplasma PCR with primers targeting the 16S rRNA-encoding locus [P1/Tint^8^, F2nR2^31^ (direct) PCR, or P1/P7^32, 33^ followed by F2nR2 (nested) PCR], as well as *cpn60*-targeted universal phytoplasma primers^12^. Many of the samples, particularly the blueberry, blackberry, and raspberry, were difficult to amplify, requiring dilution optimization in order to generate a PCR product (Fig. 1). A dilution of 1:50 was chosen for all templates analyzed by conventional PCR. Most of the strawberry samples from 2014 were negative using the 16S-targeted P1/Tint assay, although other tissues from these plants were positive by the F2nR2 direct and *cpn60*-targeted assays (Table 1). Samples from 2015 (all plants) were therefore assayed using F2nR2 (direct and nested) and *cpn60*-targeted assays.

**Table 1:** Name, origin and phytoplasma status of the tissue samples analyzed in this study.

Symptomatic Samples^a^	Tissue	Sampled in^b^	Phytoplasma status		
			PCR^c^		
			F2nR2^d^	*cpn*60 UT	LAMP^e^
**Strawberry**					
2014					
S26b-GP-MA	Green petal	M-A	+	+	+
S26b-P-MA	Fruit peduncle	M-A	NT^f,g^	NT	+
S26b-F-MA	Fruit pulp	M-A	NT^g^	NT	+
S27b-GP-MA	Green petal	M-A	+	+	+
S31b-L-MA	Midrib of green leaves with red margin	M-A	+	+	+
S31b-GP-MA	Green petal	M-A	NT^g^	+	+
S31b-P-MA	Fruit peduncle	M-A	NT^g^	NT	+
S31b-F-MA	Fruit pulp	M-A	NT^g^	NT	+
S267-GP-MA	Green petal	M-A	NT^g^	−	−
S267-P-MA	Fruit peduncle	M-A	NT^g^	NT	−
S267-F-MA	Fruit pulp	M-A	NT^g^	NT	−
S289-L-MA	Midrib of green leaves with red margin	M-A	+	+	+
S289-GP-MA	Green petal	M-A	NT^g^	NT	−
S289-P-MA	Fruit peduncle	M-A	NT^g^	+	+
S289-F-MA	Fruit pulp	M-A	NT^g^	NT	−
2015					
S01-L-MA	Midrib of green leaves with red margin	J-A	−	−	−
S01-P-MA	Fruit peduncle	J-A	+	+	+
S02-P-JA	Fruit peduncle	J-A	−	−	−
S03-P-JB	Fruit peduncle	J-B	−	−	−
S04-L-MB	Midrib of green leaves with red margin	M-B	+	+	+
S05-L-MB	Midrib of green leaves with red margin	M-B	+	+	+
S05-P-MB	Fruit peduncle	M-B	+	+	+
S06-L-MB	Midrib of green leaves with red margin	M-B	+	+	+
S06-P-MB	Fruit peduncle	M-B	+	−	+
S07-P-JC	Fruit peduncle	J-C	+	+	+
S08-L-JA	Midrib of green leaves with red margin	J-A	−	−	−
S09-L-MB	Midrib of green leaves with red margin	M-B	+	+	+
S09-P-MB	Fruit peduncle	M-B	+	+	+
S10-L-JC	Midrib of green leaves with red margin	J-C	+	+	+
S10-P-JC	Fruit peduncle	J-C	+	+	+
S11-L-JC	Midrib of green leaves with red margin	J-C	+	+	+
S12-P-JC	Fruit peduncle	J-C	+	+	+
S13-L-JC	Midrib of green leaves with red margin	J-C	+	+	+
S14-P-JA	Fruit peduncle	J-A	−	−	−
S15-L-JA	Midrib of green leaves with red margin	J-A	−	−	+
S16-P-JA	Fruit peduncle	J-A	−	−	−
S17-P-JA	Fruit peduncle	J-A	−	−	−
S18-P-JA	Fruit peduncle	J-A	−	−	−
S19-L-JD	Midrib of green leaves with red margin	J-D	+	+	+
S19-P-JD	Fruit peduncle	J-D	+	+	+
S20-L-JD	Midrib of green leaves with red margin	J-D	−	−	−
S21-L-MB	Midrib of green leaves with red margin	M-B	+	+	+
S21-P-MB	Fruit peduncle	M-B	+	+	+
S22-L-MB	Midrib of green leaves with red margin	M-B	+	−	+
S22-P-MB	Fruit peduncle	M-B	+	+	+
S23-L-MB	Midrib of green leaves with red margin	M-B	+	+	+
S24-P-JB	Fruit peduncle	J-B	−	−	−
S25-L-JC	Midrib of green leaves with red margin	J-C	+	+	+
S25-P-JC	Fruit peduncle	J-C	+	+	+
S26-L-JA	Midrib of green leaves with red margin	J-A	+	+	+
S26-P-JA	Fruit peduncle	J-A	−	−	+
S27-P-JA	Fruit peduncle	J-A	−	−	−
S28-L-MB	Midrib of green leaves with red margin	M-B	+	+	+
S29-L-MB	Midrib of green leaves with red margin	M-B	−	−	−
S30-P-MB	Fruit peduncle	M-B	−	−	−
S31-L-MB	Midrib of green leaves with red margin	M-B	+	+	+
S32-L-MB	Midrib of green leaves with red margin	M-B	+	+	+
S33-L-MB	Midrib of green leaves with red margin	M-B	+	+	+
S34-L-MC	Midrib of green leaves with red margin	M-C	−	−	−
S35-L-MD	Midrib of green leaves with red margin	M-D	−	−	+
S36-L-ME	Midrib of green leaves with red margin	M-E	−	−	+
S37-P-MD	Fruit peduncle	M-D	−	−	−
S38-L-MF	Midrib of green leaves with red margin	M-F	−	−	+
S39-L-MG	Midrib of green leaves with red margin	M-G	−	−	−
S40-L-MH	Midrib of green leaves with red margin	M-H	−	−	−
S41-L-JB	Midrib of green leaves with red margin	J-B	+	+	+
S41-P-JB	Fruit peduncle	J-B	+	+	+
S42-L-JB	Midrib of green leaves with red margin and Fruit peduncle	J-B	+	+	+
S43-L-JB	Midrib of green leaves with red margin	J-B	+	+	+
**Periwinkle**					
2014					
P83-L-SLP	Midrib of green leaves	SLP-A	+	+	+
P86-L-SLP	Midrib of green leaves	SLP-A	+	+	+
**Raspberry**					
2015					
R01-L-MA	Green leaves	M-A	+	+	+
R01-P-MA	Fruit peduncle	M-A	+	+	+
R02-L-MA	Green leaves	M-A	−	+	+
R03-L-MA	Green leaves	M-A	−	+	+
R04-L-MB	Green leaves	M-B	−	−	+
R05-L-MC	Green leaves	M-C	+	+	+
R06-L-MC	Green leaves	M-C	−	+	+
R07-L-MC	Green leaves	M-C	−	−	+
R08-L-MC	Green leaves	M-C	+	+	+
**Blueberry**					
2015					
Bl01-L-JA	Yellow leaves	J-A	+	+	+
Bl02-L-JB	Leaves showing red margin	J-B	−	−	+
**Blackberry**					
2015					
Bk01-L-JC	Green leaves	J-C	−	+	+
Bk02-L-JD	Green leaves	J-D	+	+	+
Bk03-L-MD	Green leaves	M-D	−	+	+
Bk04-L-ME	Green leaves dry	M-E	−	−	−

^a^Symptoms observed in strawberry and periwinkle plants were previously described^30^. Blackberry and raspberry plants showed small leaves and green structures in the fruits; blueberry plants showed yellow leaves and leaves with red margins (Figure S1). ^b^Michoacan samples: 20.002°N, 102.3089°W; San Luis Potosi samples: 22.2°N, 100.1°W; Jalisco samples: 20.34°N 103.41°W. A total of 11 commercial farms located among the three states mentioned above were visited to collect the symptomatic samples. ^c^PCR using primers R16F2n/R16R2^31^ to amplify the F2nR2 sequence and H279p/H280p^12^ to amplify the *cpn*60 UT sequence. ^d^Nested PCR was performed on the samples collected in 2015, with P1/P7 primers in the first reaction^32, 33^, and R16F2n/R16R2 in the nested reaction^31^. Samples collected in 2014 were analyzed with direct PCR targeting the F2nR2 fragment. ^e^LAMP using primers and conditions described in Table 1. ^f^NT, not tested. ^g^Sample was negative using 16S-targeted primers P1/Tint^8^.

We compared the performances of the 16S-targeted F2nR2-nested and the direct *cpn60*-targeted conventional PCR assays. A total of 69 samples including 54 from strawberry, nine from raspberry, two from blueberry and four from blackberry were included in the analysis. 38 samples were positive by F2nR2-nested PCR and 31 were negative. Of the positives, the ~605 bp *cpn*60 UT amplicon was generated from 36 samples. The sensitivity of the *cpn60*-targeted phytoplasma PCR was high (95%) when compared to the F2nR2-nested PCR (Table 2), which is consistent with a low false negative rate. The specificity of the *cpn60* UT-targeted conventional PCR assay was 87%.

**Table 2:** Comparison of the performances of the direct *cpn60* UT-targeted and nested PCR assay targeting F2nR2.

*cpn60* UT PCR results	F2nR2 nested PCR results		Total	
	Positive	Negative		
**Positive**	36	4	40	
**Negative**	2	27	29	
**Total**	38	31	69	
	95%CI	Low	High	
**Test sensitivity**	0.947	0.071	0.876	1.018
**Test specificity**	0.871	0.118	0.753	0.989

Discordant samples were analyzed for the presence of SbGP/MPV phytoplasma DNA. Of the eleven samples that were negative by F2nR2 direct or nested PCR but positive with the direct *cpn60*-targeted conventional PCR assay, all were positive with the *cpn60* UT-targeted LAMP and quantitative PCR assays (Supplementary Table S1). Furthermore, *cpn60* UT sequences were successfully determined for five of these samples, which all showed 100% identity to the SbGP/MPV phytoplasma sequence. These results demonstrated that the *cpn60*-targeted direct PCR performed well compared to the F2nR2-targeted PCR in either format (nested and direct), and that the F2nR2 nested PCR generated more positives compared to the direct PCR (Table 1).

Two samples were identified as false negatives using the *cpn60* UT-PCR assay (Supplementary Table S1). These samples were positive by F2nR2 PCR (direct and nested), and were also positive using the *cpn60* UT-targeted LAMP and qPCR assays (Supplementary Table S1). Also, one of these samples (S22-L-MB) was positive with the fluorescent microsphere hybridization assay (Supplementary Table S1), which uses essentially the same amplification primers.

### Expanded oligonucleotide-coupled fluorescent microsphere hybridization assay for phytoplasmas

**Figure 2:**
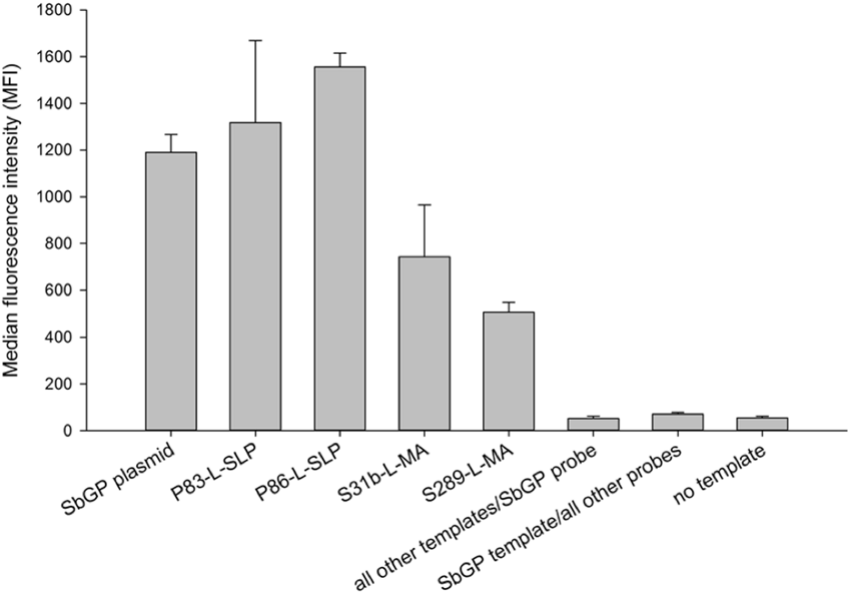
Representative median fluorescence intensities observed using the fluorescent microsphere hybridization assay on *cpn60* UT amplicon generated from strawberry and periwinkle DNA templates. “All other templates” are *cpn60* UT amplicons generated from AY-Ruta (16SrI-A, ‘*Ca*. P. asteris’- related strain), SF1 (16SrI-B, ‘*Ca*. P. asteris’-related strain), CVB, AY-Col (16SrI-C, ‘*Ca*. P. asteris’-related strain), RS (16SrV-A, ‘*Ca*. P. ulmi’- related strain), AshY (16SrVII-A, ‘*Ca*. P. fraxini’-related strain), Cr (16SrIX-H, ‘*Ca*. P. phoenicium’-related strain), AP (16SrX-A, ‘*Ca*. P. mali’-related strain), PYLR (16SrX-C, ‘*Ca*. P. pyri’-related strain), ESFY (16SrX-F, ‘*Ca*. P. prunorum’-related strain), and BN44948 (16SrXII-A, ‘*Ca*. P. solani’-related strain). “All other probes” are the probes that specifically target these strains, as previously described^12^.

The probe for SbGP/MPV phytoplasma (Supplementary Table S2) detected only the target sequence among all of the phytoplasma *cpn60* UT sequences that have been reported by our group (Fig. 2). Strong fluorescence signals were observed in the samples consisting of either the cloned SbGP/MPV *cpn60* UT plasmid DNA or genomic DNA samples derived from symptomatic strawberry and periwinkle plants (Fig. 2). The average of the MFI readings for all of the other 11 probes using the SbGP *cpn60* UT plasmid or genomic templates was not significantly greater than that observed with no template; similarly, no significantly positive MFI signal was observed when the SbGP/MPV probe was used on all of the other templates. These results confirm both that the SbGP/MPV probe reacted only with the desired target DNA (among those tested), and that no other template DNA (among those tested) generated a signal with the SbGP/MPV probe. Applying the fluorescent microsphere hybridization assay to the strawberry samples from 2015 revealed a good correspondence with the results of the *cpn60* UT-targeted conventional PCR (Supplementary Table S3). In all cases but one, only the SbGP/MPV probe generated a significantly positive MFI result, demonstrating the capacity of this assay to detect and simultaneously type phytoplasma infections.

Samples S41-L-JB and S41-P-JB, both from the same strawberry plant, showed no significant signal for SbGP/MPV probe, but a significantly positive MFI signal was observed for the AY-SF1 probe^12^ (16SrI) (Supplementary Table S3). These samples were positive for SbGP/MPV phytoplasma by LAMP and qPCR targeting *cpn60* UT (Supplementary Table S3). To determine if this sample contained DNA from both AY and SbGP/MPV phytoplasmas, sequences from individual F2nR2 clones generated from sample S41-L-JB were examined. Phylogenetic analysis of these sequences revealed that one clone clustered with 16SrI-B phytoplasma (KY061166). This clone had >99% nucleotide sequence similarity with MBS phytoplasma (AY265208). Another clone was identified as deriving from SbGP/MPV phytoplasma [16SrXIII-I (KY061167; Supplementary Fig. S1)]. Furthermore, the *cpn60* UT sequence obtained from this plant was identified as *cpn60* UT I-IIIB (KY061183) subgroup phytoplasma^18^. A total of 8 clones were examined, all of which corresponded to the AY *cpn60* UT I-IIIB subgroup (not shown).

### Quantification of SbGP/MPV and AY phytoplasma in plant tissues using qRT-PCR

**Figure 3:**
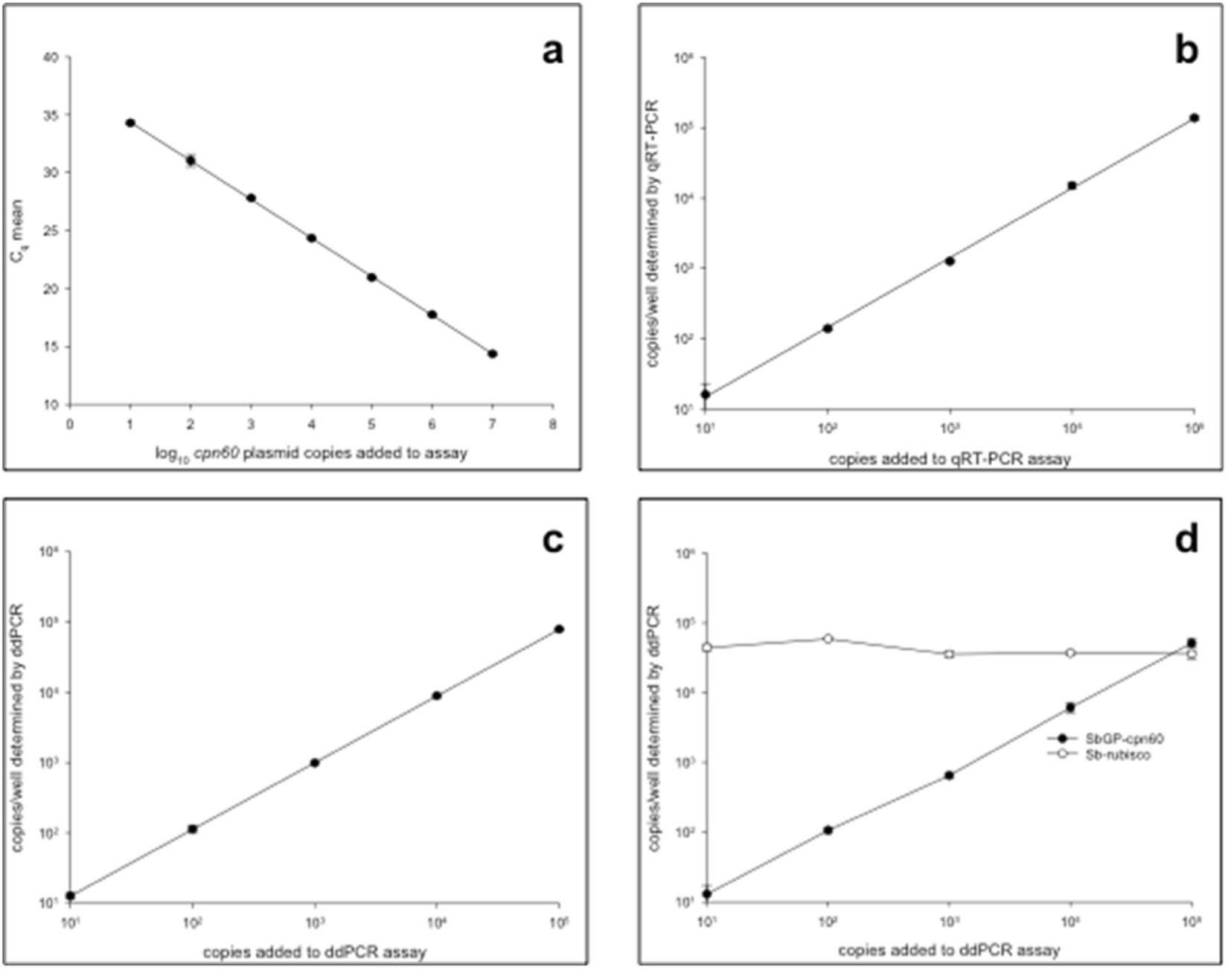
(**a**) qRT-PCR standard curve for *cpn60*-targeted SbGP detection assay. PCR efficiency (E) was determined to be >1.99 using E = 10^(−1/slope)^ ^32^. Results shown are the means of duplicate determinations ± standard deviation. (**b**,**c**,**d**) Quantitative PCR assay (**b**), qRT-PCR; (**c**,**d**), ddPCR) accuracy, linearity, and detection limits determined by relating the number of copies of SbGP *cpn60* UT plasmid DNA added to the reaction to the number of copies measured. ddPCR assays were performed in the absence (**c**); 1-plex) and presence (**d**); 2-plex) of DNA extracted from a healthy strawberry plant. qRT-PCR assays were performed in the presence of uninfected strawberry DNA. Results shown are the means of duplicate measurements ± standard deviation.

Dilution optimization for the qRT-PCR assay indicated that PCR inhibition was not a significant factor for detecting and accurately quantifying the target DNA in these samples, as all dilutions analyzed gave similar results (Supplementary Fig. S5). The assay that was implemented for qRT-PCR detection of SbGP/MPV was highly efficient using plasmid standards (Fig. 3a), with a mean PCR efficiency (E) of 1.991 ± 0.005 (n = 3), where 2.0 is theoretical^34^. In addition, the assay was highly linear (correlation of >0.999 between copies added and copies detected) and accurately determined the number of copies of SbGP/MPV *cpn60* in spiked strawberry samples (mean accuracy of 1.42 over 10 samples spanning 5 orders of magnitude; Fig. 3b). The qRT-PCR assay consistently detected samples with as few as 10 copies of SbGP/MPV per assay (Fig. 3a,b). Examining the strawberry and periwinkle samples harvested in 2014, the qRT-PCR assay revealed that nearly all samples analyzed contained very high levels of SbGP/MPV phytoplasma DNA, and that the periwinkle samples contained higher levels than any of the strawberry samples (Tables 3 and S3). The qRT-PCR assay failed to generate a signal using healthy strawberry or periwinkle DNA, and none of the nontarget phytoplasma *cpn60* UT plasmids were positive in this assay (Table 3). The strawberry samples from 2014 generally contained high levels of SbGP/MPV phytoplasma in all tissues, particularly in the leaves and fruit peduncle (Table 3). One of the samples, S289-MA, showed high levels of SbGP/MPV phytoplasma DNA in the leaf and fruit peduncle but undetectable levels in the green petal and fruit pulp, consistent with observations made by ddPCR and LAMP (Table 3). Another sample, S267-MA, showed very low levels of SbGP/MPV phytoplasma DNA in the green petal only using qRT-PCR (consistently positive over 4 observations), yet this sample was negative by ddPCR and by LAMP (Table 3).

**Table 3:** Molecular quantification of SbGP/MPV phytoplasma in infected plant samples obtained in 2014.

Sample	Method Unit	qRT-PCR genomes/g tissue		ddPCR genomes/g tissue		LAMP Time to positive (Tp), minutes (calcein detection)	
	Tissue	Mean (n)	Standard deviation	Mean (n)	Standard deviation	Mean (n)	Standard deviation
S31b-L-MA	Leaf midrib	4.82 × 10^8^ (10)	5.48 × 10^7^	2.41 × 10^8^ (10)	2.51 × 10^7^	45.88 (2)	3.01
S31b-GP-MA	Green petal	2.74 × 10^9^ (2)	7.58 × 10^7^	4.80 × 10^8^ (2)	4.88 × 10^7^	31.88 (2)	3.00
S31b-P-MA	Fruit peduncle	4.34 × 10^8^ (2)	2.01 × 10^7^	2.11 × 10^8^ (2)	2.61 × 10^7^	35.0 (3)	0.43
S31b-F-MA	Fruit pulp	9.69 × 10^7^ (2)	1.80 × 10^7^	5.48 × 10^7^ (2)	4.24 × 10^6^	45 (2)	3.54
S289-L-MA	Leaf midrib	1.34 × 10^8^ (10)	1.78 × 10^7^	7.94 × 10^7^ (9)	1.14 × 10^7^	49.63 (2)	3.01
S289-GP-MA	Green petal	ND^a^ (2)		ND (2)		ND (2)	
S289-P-MA	Fruit peduncle	4.24 × 10^8^ (2)	1.29 × 10^7^	7.16 × 10^7^ (2)	3.61 × 10^6^	35.38 (2)	0.18
S289-F-MA	Fruit pulp	ND (2)		ND (2)		ND (2)	
S26b-GP-MA	Green petal	1.12 × 10^9^ (2)	7.44 × 10^7^	3.85 × 10^8^ (2)	7.64 × 10^6^	35.38 (2)	1.24
S26b-P-MA	Fruit peduncle	1.04 × 10^9^ (2)	2.90 × 10^8^	4.23 × 10^8^ (2)	6.36 × 10^6^	25.25 (2)	0
S26b-F-MA	Fruit pulp	3.98 × 10^8^ (2)	1.22 × 10^7^	1.46 × 10^8^ (2)	1.09 × 10^7^	38.38 (2)	0.18
S27b-GP-MA	Green petal	4.01 × 10^9^ (2)	4.07 × 10^8^	6.92 × 10^8^ (2)	1.23 × 10^8^	30.88 (2)	1.59
S267-GP-MA	Green petal	8.21 × 10^2^ (4)	4.53 × 10^2^	ND (2)		ND (2)	
S267-P-MA	Fruit peduncle	ND (2)		ND (2)		ND (2)	
S267-F-MA	Fruit pulp	ND (2)		ND (2)		ND (2)	
P83-L-SLP	Leaf midrib	4.67 × 10^9^ (10)	7.72 × 10^8^	2.53 × 10^9^ (10)	1.80 × 10^8^	38.42 (3)	2.31
P86-L-SLP	Leaf midrib	6.13 × 10^9^ (10)	8.46 × 10^8^	2.82 × 10^9^ (10)	3.10 × 10^8^	37.08 (3)	2.13
Strawberry (H)^b^	Leaf	ND (2)		ND (2)		ND (2)	
Periwinkle (H)	Leaf	ND (2)		ND (2)		ND (2)	
Raspberry (H)	Fruit	ND (2)		ND (2)		ND (2)	
Blueberry (H)	Leaf	ND (2)		ND (2)		ND (2)	
Blackberry (H)	Fruit	ND (2)		ND (2)		ND (2)	
Nontarget^c^		ND (2)		ND (2)		ND (2)	

^a^ND, not detected. ^b^H, DNA from uninfected plant. See Supplementary Fig. S4 for results. ^c^Nontarget DNA is a mixture of plasmids consisting of 10^6^ copies each of all of the *cpn60* UT fragments from the phytoplasma strains described in Dumonceaux *et al*.^12^. See Supplementary Fig. S4 for results on individual templates.

Generally, somewhat lower levels of SbGP/MPV phytoplasma DNA were found in the strawberry samples harvested in 2015, with some samples generating C_q_ values that were below that of the lowest standard (Supplementary Table S3). Despite showing strong symptoms (Supplementary Fig. S2), the samples obtained from infected raspberry, blueberry, and blackberry also showed relatively low levels of phytoplasma DNA (Supplementary Table S3).

SbGP/MPV phytoplasma DNA was detected in the leaf and peduncle of sample S41-JB (Supplementary Table S3). Since the oligonucleotide-coupled fluorescent microsphere hybridization assay as well as cloning suggested that this plant harbored a double infection with AY and SbGP/MPV phytoplasma, we used a previously described AY (Aster yellows)- specific qRT-PCR assay^35^ to examine these samples. We also analyzed samples Bl02-L-JB, S03-P-JB, S24-P-JB, S42-L-JB, and S43-L-JB, all of which were collected in the same locality (B) in Jalisco. The AY-qRT-PCR showed that all the samples were positive using both assays. However, only the samples derived from plant S41-JB were quantifiable, with approximately 150-fold higher copy number of AY-phytoplasma compared to SbGP/MPV phytoplasma (Supplementary Table S4).

### Quantification of SbGP/MPV phytoplasma using ddPCR

The ddPCR-adapted version of the assay performed similarly to the qRT-PCR assay in serial dilutions of template, except at very low levels of target (Supplementary Fig. S5), suggesting that PCR inhibition was not a significant factor for the quantitative assays. Calibration of the ddPCR assay using known copy numbers of target *cpn60* UT plasmids revealed that the assay was highly linear; the “calibration curve” showed a correlation of >0.999 between the number of copies added and the number of copies detected (Fig. 3b). Moreover, the assay was accurate both in the absence (Fig. 3c) and presence (Fig. 3d) of DNA from uninfected strawberry plants, with a mean accuracy of 0.83 across 10 samples spanning 5 orders of magnitude. The ddPCR assay was capable of detecting samples with as few as 10 copies of SbGP per reaction consistently (Fig. 3c,d). Applying the assay to the infected plant DNA extracts showed similar results to the qRT-PCR assay when expressed in terms of phytoplasma genome copies/g tissue extracted (Tables 3 and S3). Similar trends in copy numbers were observed in the samples by ddPCR and qRT-PCR, with P86-L-SLP showing the highest copy number (Tables 3 and S3). Analyzing the reproducibility of the qRT-PCR and ddPCR assays showed that the ddPCR assay had consistently lower measurement error than was observed by qRT-PCR for samples repeated up to 10 times (Table 3; samples S31b-L-MA, S289-L-MA, P83-L-SLP, P86-L-SLP). No detection of any non-target phytoplasma template DNA was observed, nor was any signal generated using uninfected strawberry or periwinkle DNA (Table 3 and Supplementary Fig. S4). ddPCR also revealed the presence of SbGP/MPV phytoplasma in samples S41-JB (L and P) with quantifiable values (Supplementary Table S3). Application of an internal control for the strawberry samples enabled the expression of ddPCR results in terms of fractional abundance (Supplementary Table S3), which corrects for any intersample variation in DNA yield and amplifiability.

### Detection of SbGP/MPV phytoplasma using LAMP

**Figure 4:**
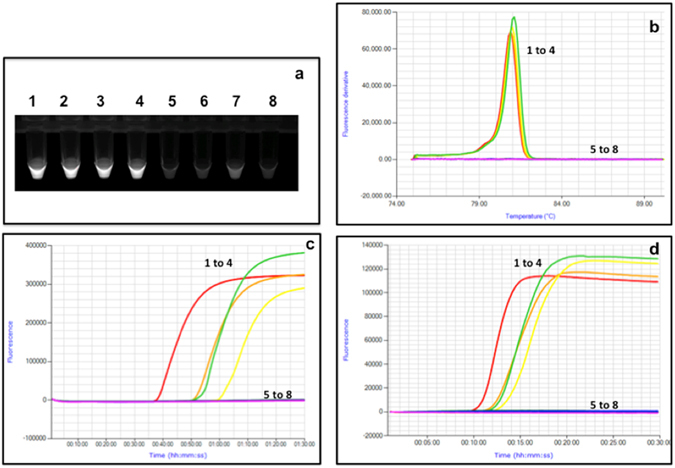
LAMP assay targeting the SbGP/MPV*cpn60* gene applied to plant DNA extracts. 1, S31b-L-MA; 2, 289-L-MA; 3, P83-L-SLP; 4, P86-L-SLP; 5, healthy strawberry DNA; 6, healthy *C. roseus* DNA; 7, mixture of 10^6^ copies each of all non-target plasmid templates (see Table 3); 8, no template control. (**a**) Reactions were viewed under ultraviolet light using a transilluminator after amplification using the Genie instrument. (**b**,**d)** Annealing and Amplification curves respectively, for the same samples using Isothermal detection chemistry. (**c**) Amplification curves for the same samples using calcein-based detection method.

Like the qRT-PCR and ddPCR assays, the LAMP assay was negative using DNA from uninfected host plant tissue as well as nontarget phytoplasma *cpn60* UT plasmid DNA (Table 3, Fig. 4, and Supplmental Fig. S4). The LAMP assay afforded rapid detection of SbGP/MPV phytoplasma DNA, with positive results observed in as little as 25 minutes using a calcein-based detection system (Supplementary Table S3). To analyze the effect of PCR inhibitors on the detection of SbGP/MPV phytoplasma DNA by LAMP, serial dilutions of samples S05-L-MB, Bl01-L-JA, R05-L-MC, and Bk02-L-JD were examined. Nearly all dilutions, including those that were negative by conventional PCR targeting 16S and *cpn60* UT, were positive by LAMP (Fig. 1). Other samples were also shown to amplify robustly across dilutions, like the quantitative PCR-based assays (Supplementary Fig. S5). The LAMP assay also showed an inverse relationship between input copy number and T_p_ that was linear over several orders of magnitude (Supplementary Fig. S3), with the isothermal detection chemistry affording much more rapid detection with lower measurement error. The LAMP assay in both formats could consistently detect as few as 100 copies of SbGP/MPV phytoplasma DNA, although the calcein-based detection system required slightly more than 60 minutes (the defined assay time) to detect this amount (Supplementary Fig. S3).

Using calcein detection chemistry, the 2015 samples were analyzed (undiluted and 1:50 dilutions) with a binomial system by viewing the reactions under ultraviolet light at the end of the assay (e.g. Fig. 4a). The binomial system eliminated the quantitative aspect of the assay as samples with higher levels of SbGP/MPV phytoplasma DNA looked no different from samples with lower levels (Fig. 4a). Using this system, all samples that tested positive for phytoplasma by F2nR2 nested PCR were positive by LAMP, suggesting a perfect concordance on positive samples (Supplementary Table S3). Moreover, 10 symptomatic samples that were F2nR2 negative using conventional PCR were positive using the *cpn60* UT-targeted LAMP assay.

The 2015 samples from berry plants were analyzed using the same LAMP primers, but with Isothermal detection chemistry. This facilitated the determination of the time to positive (T_p_) for each sample along with a diagnostic annealing temperature that is characteristic of the specific LAMP product generated. The T_p_ of these samples were very low, with positive results observed in less than 10 minutes in many cases (Supplementary Table S3). The annealing temperature of the SbGP/MPV LAMP product was approximately 81 °C (Supplementary Table S3). The concordance between LAMP results detected using calcein and isothermal detection chemistry was generally very good, although 7 of the 69 samples were positive by calcein and negative by isothermal chemistry while one sample (S36-L-ME) was positive using isothermal chemistry but negative using calcein. All raspberry, blueberry, and blackberry samples analyzed showed perfect agreement between the two detection methods (Supplementary Table S3).

### Geographic distribution of SbGP/MPV phytoplasma in central Mexico

**Figure 5:**
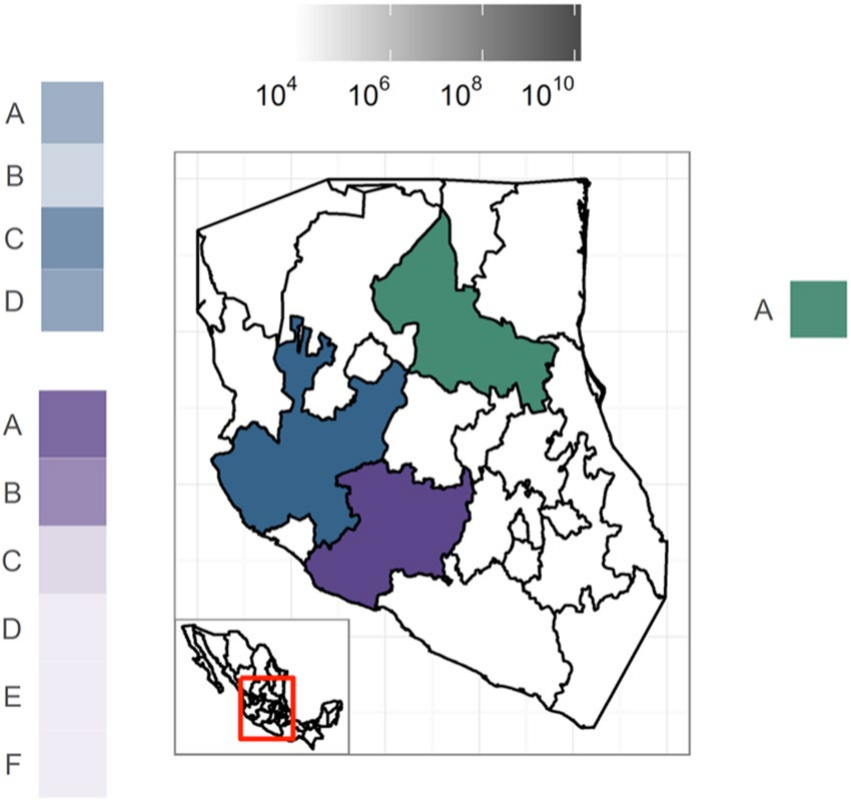
Geographic distribution of SbGP/MPV phytoplasma per farm in central Mexico. Samples from San Luis Potosi (green), Michoacan (blue), and Jalisco (purple) states were examined using qRT-PCR targeting *cpn60*. Samples from each locality within each state (A-F) are shaded according to the mean number of copies of SbGP/MPV phytoplasma *cpn60* per g tissue extracted. To generate the map, data was accessed from the GADM database (Global Administrative Areas 2012). GADM database of Global Administrative Areas, version 2.0. [online] URL: www.gadm.org) and plotted in R (http://www.R-project.org/) using ggplot2^59^.

Taken together, the provenance of all samples and the results of the quantitative molecular diagnostic assays showed that SbGP/MPV phytoplasma is widespread over an area covering three Mexican states, which range in size from ~60,000 (Michoacan and San Luis Potosi) −80,000 km^2^ (Jalisco) (Fig. 5). SbGP/MPV phytoplasma was detected in multiple locales in two of these states, with 11 localities represented in the three states. Examination of the quantitative data revealed that samples from some localities tended to show higher levels of SbGP/MPV phytoplasma than others; for example samples from locations A and B within Michoacan were notably higher than samples from other localities in this state (Fig. 5).

## Discussion

Symptomatic plants collected from different geographic areas in Mexico yielded variable results with the pan-phytoplasma PCR depending on the tissue sampled, with positive results generated from leaves and leaf midribs but negative results from floral parts and fruits (Table 3, Supplementary Table S3). It is well known that the high concentration of phenolic compounds in strawberry and other berry fruits^36^ can inhibit PCR^37^, which likely explains why DNA extracted from symptomatic plants using petals, fruit peduncle or fruit as starting material were initially PCR negative in many cases. Analysis using the more inhibitor-tolerant LAMP assay revealed that most of these samples were positive for SbGP/MPV phytoplasma DNA (Table 3, Supplementary Table S3). This suggests that leaves from symptomatic plants are a preferred starting material for initial screening using conventional PCR targeting all phytoplasmas.

A suite of molecular diagnostic assays that target the single-copy *cpn60* gene of the SbGP/MPV phytoplasma was designed. Rapid, sequencing-independent detection and typing of phytoplasma infection was provided by the fluorescent microsphere hybridization assay. These results expand the previously reported 11-plex *cpn60*-based phytoplasma detection array^12^ to 12-plex, demonstrating the flexibility of this assay format. The assay was also found to be semi-quantitative (Fig. 2 and Table 3), although as an end-point PCR-based method MFI would not be expected to correlate to copy number over a wide dynamic range. The quantitative assays showed very high levels of SbGP/MPV DNA in some of the plant samples, with up to 10^9^ phytoplasma genomes/g tissue in the leaf samples analyzed. Both of the quantitative PCR-based assays gave similar results, but the ddPCR assay had a lower measurement error and appeared to be highly accurate in its determination of the copy number of SbGP/MPV *cpn60* genes in the sample. Combined with the fact the ddPCR appears to be more resistant to the effects of PCR inhibitors compared with qRT-PCR^38^, this indicates that ddPCR may generally be a preferred method for highly accurate quantification of SbGP/MPV phytoplasma genomes in plant DNA extracts. However, this must be countered by the fact that the ddPCR assay is longer, with more operator steps involved, and is more expensive than qRT-PCR, which gives essentially identical results. Moreover, ddPCR has a lower dynamic range than qRT-PCR, which means that strongly positive samples such as some of those sampled here require optimization of the dilution to get the sample into the quantitative range. The use of internal controls in ddPCR requires additional optimizations of the technique to find the balance between detectable phytoplasma and measurable internal control, although it provides the benefit of normalizing the detected pathogen DNA to a known quantity of host DNA and thereby corrects for variability in DNA yields among samples. The choice of quantitative assay therefore depends on instrument availability and operator preference.

Rapid and field-deployable detection of pathogen DNA in samples is a key advantage of the use of LAMP for phytoplasma detection^39^. LAMP assays targeting many different phytoplasma groups have been described^40, 41^, some of which target *cpn60* ^17^. However, no LAMP assays targeting SbGP/MPV phytoplasmas (16SrXIII) have been described to date. The SbGP/MPV-*cpn60* UT-targeted LAMP assay we described was rapid, offering positive results in as little as 9 minutes (isothermal detection chemistry) on strongly positive samples. While isothermal detection chemistry was rapid, linear, and provided a diagnostic annealing temperature for the product, it is more expensive than other detection systems and requires a dedicated instrument to visualize the results. The instrument we used (Genie) is ideally suited for field-based applications, but it is expensive and not necessarily suited to resource-limited settings. However, the LAMP assay equally provides binomial results that are viewable under ultraviolet light when calcein is used as the detection reagent, or under visible light with other detection schemes such as hydroxy naphthol blue^42^. Like ddPCR, LAMP is typically seen as more resistant to the effects of PCR inhibitors compared to other, PCR-based detection methods^43^, and LAMP is also normally considered to have an analytical sensitivity that is equal to or better than PCR-based methods^44^. This fact likely explains why many PCR-negative samples were positive using the LAMP assay. The LAMP assay we have described is likely to detect SbGP/MPV phytoplasma DNA in most symptomatic plant tissues, and probably in positive insects as well since insects accumulate high levels of phytoplasma in the salivary glands^6^.

The molecular diagnostic assays we employed facilitated the detection of a double infection of some samples with AY and SbGP/MPV phytoplasma. Double infections are not unusual in plants affected by phytoplasmas. Aster yellows phytoplasma and flavescence dorée phytoplasma have been detected affecting the vector *Euscelidius variegatus* in Italy^45^, and also in grapevine plants^46^. In fact, this study is not the first to find phytoplasmas belonging to the aster yellows and Mexican periwinkle phytoplasma groups affecting the same host. Broccoli plants in Brazil were affected by strains from these groups, as well as by phytoplasmas belonging to the 16SrIII ribosomal group^47^. Usually, the strategy followed to address double infections requires the sequencing of a large number of F2nR2 clones along with the sequencing of a protein-encoding gene^30, 47^. In this study the double infection was detected through the complementarity of the different SbGP/MPV phytoplasma-*cpn*60-based molecular diagnostic methods developed, along with previously developed AY phytoplasma *cpn*60-based specific diagnostic methods. Based on the quantitative results obtained through SbGP/MPV- qRT-PCR and AY- qRT-PCR of samples S41-JB, at least 150 clones would need to be sequenced to identify SbGP/MPV phytoplasma through the sequencing of *cpn60* UT clones, which is a laborious and expensive process. The identification of aster yellows in plants affected by strawberry green petal disease is not surprising, since this disease is typically associated with phytoplasmas of the aster yellows (‘*Ca*. Phytoplasma asteris’, 16SrI) group^19–22, 48^.

We compared the performance of the *cpn60*-targeted direct PCR assay to nested PCR targeting F2nR2. A wide variety of PCR primer combinations has been reported in the literature for the detection of phytoplasmas^8, 49^, and we previously compared the *cpn60*-targeted direct PCR^12^ to direct PCR using primers P1/Tint^8^. More recently, 16S rRNA-encoding gene-targeted primers have been described that are suitable for both conventional and real-time PCR^50^, but these have not been demonstrated to work with the 16SrXIII group. We therefore chose to compare the *cpn60*-targeted direct PCR assay to nested PCR targeting the F2nR2 nested PCR, which is commonly used to detect phytoplasma infections. The clinical sensitivity of the *cpn60* UT-targeted pan-phytoplasma PCR compared to F2nR2-nested PCR was similar to its previously reported sensitivity compared to the P1/Tint-PCR assay^12^, but the specificity observed was higher (87.1% compared to F2nR2; 44.4% by comparison to P1/Tint^12^). These results suggest that P1/Tint primers may have a higher rate of false negative results compared to the phytoplasma *cpn60* UT-targeted primers and primers P1/P7 and R16F2n/R16R2. Moreover, the band amplified by P1/Tint does not always correspond to phytoplasma^51^. The identification of the “false positives” as real positives was achieved through the different SbGP/MPV phytoplasma *cpn60* UT-based specific molecular diagnostic methods developed in this study, reinforcing the application of the array of *cpn60* UT-based diagnostics presented here.

In this study, blueberry, blackberry, and raspberry plants were identified for the first time as hosts for phytoplasmas in Mexico, and also as hosts for 16SrXIII phytoplasma group worldwide. Recently, two species of ‘*Candidatus* Phytoplasma’ have been described among the members of the 16SrXIII group, ‘*Ca*. Phytoplasma hispanicum’^28^ and ‘*Ca*. Phytoplasma maliae’^52^ which have been reported affecting periwinkle^27^, strawberry^24, 26^, potato^29^, papaya^53^, broccoli^47^, and china tree^54^ plants. Among the berry plants analyzed in this study, only strawberry has previously been identified as a host for SbGP/MPV phytoplasma.

Our results also show the minimum extent of the geographic distribution of SbGP/MPV phytoplasma in Central Mexico. One weakness of the study is the lack of the exact position of each farm sampled, but we have demonstrated that the results obtained through the quantitative diagnostic methods can be used to assess the distribution of the phytoplasma in defined geographic areas. Although our study selected symptomatic samples, with an alternative sampling strategy the prevalence of this disease could be easily monitored using the molecular diagnostic assays we describe. The molecular diagnostic assays described in this study can provide timely, accurate information about the identification, characterization, geographic distribution and prevalence of this phytoplasma in plant and insect hosts within the Americas and in other geographic areas.

## Materials and Methods

### Plant material

Tissues from symptomatic strawberry (*Fragaria x ananassa*) and periwinkle (*Catharanthus roseus*)^30^, along with raspberry (*Rubus idaeus*), blueberry (*Vaccinium corymbosum*), and blackberry (*Rubus fructicosus*) plants with symptoms described in Table 1 and Figure S1 were analyzed. Samples were collected in one locality (A) in San Luis Potosi, four localities in Jalisco (A-D), and 6 localities in Michoacan (A-F) across two growing seasons (November-February), 2014 and 2015. Samples were coded according to the plant, tissue, state, and locality from which they were obtained (Table 1). In total, 69 samples from 48 strawberry plants, 2 samples from 2 periwinkle plants, 9 samples from 8 raspberry plants, 2 samples from 2 blueberry plants, and 4 samples from 4 blackberry plants were collected. DNA was extracted from these samples as previously described^51^.

### Conventional PCR and cloning of PCR products

PCR primers P1/P7^32, 33^ and R16F2n and R16R2^31^, which generate the ~1.2 kb F2nR2 amplicon from all phytoplasmas, were used to amplify the 16S rRNA-encoding locus of the phytoplasma-infected samples analyzed in this study. The primers were used either in a “nested” format with the first step using P1/P7 and the second step using R16F2n/R16R2, or a “direct” format using only the latter primers. For the nested format, the initial PCR (P1/P7) amplified a >1.8-kbp product, which was diluted (1:30) (Supplementary Fig. S6), and used as template in a secondary PCR step with primers R16F2n and R16R2 (Supplementary Fig. S6). In addition, PCR primers designed to amplify the ~600 bp *cpn60* UT of all phytoplasmas^12^ were used to generate *cpn60* UT amplicon from the same samples. The sensitivity and specificity of the *cpn60* UT primers were determined using the nested F2nR2 amplification as to define positives and negatives as described^55^.

The F2nR2 amplicon generated from samples S07-P-JC and S10-L-JC was cloned into the vector pGEM-T Easy (Promega, Madison, WI USA) according to the manufacturer’s recommendations, then plasmids were transformed into chemically competent *E. coli* TOP10 (Life Technologies), and five and three clones were sequenced using plasmid-targeted primers T7/SP6, respectively. The *cpn60* UT amplicons from the same samples were generated and sequenced directly with the primers H0279p/H0280p as previously described^12^. The *cpn60* UT from samples S05-L-MB (strawberry), Bl01-L-JA (blueberry), R05-L-MC (raspberry), and Bk02-L-JD (blackberry), were amplified and sequenced directly to confirm the presence of phytoplasmas and identify the phytoplasma affecting the plants. The F2nR2 and the *cpn60* UT amplicons generated from sample S41-L-JB were also cloned and sequenced as described above.

### Detection and identification of SbGP/MPV phytoplasma using oligonucleotide-coupled microspheres

Methods for probe design and amplicon generation and hybridization have been described in detail^12, 56^. Briefly, oligonucleotides that distinguish SbGP/MPV phytoplasma *cpn60* UT from other phytoplasma *cpn60* UT sequences (Supplementary Table S2) were designed using sigoligo^57^ and PrimerPlex v2.62 (Premier Biosoft, Palo Alto CA). Oligonucleotides were coupled to fluorescent microspheres and hybridized to single-stranded PCR product generated using the *cpn60* UT-targeted universal primers for phytoplasma^12^. Samples with median fluorescence intensities (MFI) that were significantly greater than controls were identified as positive, using duplicate hybridizations and a one-tailed student’s t-test with a significance cut off of 0.05.

### Molecular diagnostic assay based on qRT-PCR detection of the SbGP/MPV *cpn60*

Regions of the SbGP/MPV *cpn60* UT that distinguished the target gene from all other described *cpn60* UT sequences of ‘*Ca*. Phytoplasma’ spp. were identified using sigoligo^57^. These regions were chosen for hydrolysis probe assay design using Beacon Designer v.7.90 (Premier Biosoft, Palo Alto, CA). A primer/probe set was selected based on primer BLAST that was likely to selectively target SbGP/MPV phytoplasma (Supplementary Table S2). All amplification primers and the hydrolysis probe were obtained from Integrated DNA technologies (Coralville, IA). The primer/probe set was evaluated for analytical specificity using 10 ng of template DNA obtained from uninfected strawberry (obtained from a local grocery store) and periwinkle (obtained from control plants maintained in a growth chamber), and a mixture of plasmid DNA consisting of 10^6^ copies each of all of the phytoplasma *cpn60* UT sequences previously described^12^. The final amplification conditions are shown in Supplementary Table S2. For quantification, a set of standards was prepared using plasmid DNA containing the *cpn60* UT of SbGP/MPV phytoplasma. Miniprep DNA prepared using a Qiagen miniprep kit (500 ng) was linearized using *Pst*I in a 50 µl digestion volume, then purified using a QiaQuick column (Qiagen). The concentration of linearized, purified plasmid DNA was determined in triplicate using a Qubit instrument (BR kit, Life Technologies), and the mean concentration (ng/µl) was converted to copies/µl using the known length of the plasmid DNA and an approximation of 650 g/mol per base pair. Standards were diluted to concentrations of 10^7^ − 10^1^ copies per 2 µl and used as control templates in qRT-PCR. Reactions were prepared using SsoFast Universal probes supermix (Bio-Rad, Mississauga, ON, Canada) in a 20 µl final volume with 300 nM of each primer and 200 nM of probe. Amplification was carried out using a C1000 thermocycler base with a CFX96 real-time system (Bio-Rad) and reactions were quantified using BioRad CFX manager software (v.3.1). Threshold cycle (C_q_) values were converted to copy numbers by interpolation on the standard curve, and the results were corrected to account for sample preparation and dilution to arrive at copy number/g tissue extracted. Positive samples with C_q_ values below that of the lowest standard were specified as, “detectable but not quantifiable” (DNQ).

### ddPCR quantification of SbGP/MPV *cpn60*

The same primer/probe set used for the qRT-PCR assay was used for ddPCR quantification. Reaction conditions were optimized using gradient PCR with ddPCR supermix for probes (Bio-Rad) including 900 nM each primer and 250 nM of hydrolysis probe in a 20 µl reaction volume with a plasmid standard prepared as described above (10^3^ copies per assay). Final ddPCR conditions are shown in Supplementary Table S2. Template DNA extracted from strawberry and periwinkle samples analyzed by ddPCR was digested prior to amplification using *Eco*RI at 37 °C for 60 minutes followed by enzyme inactivation at 85 °C for 5 minutes. Samples were then diluted 1:100 and 2 µl used as template for ddPCR. Emulsions were formed using a QX100 droplet generator (Bio-Rad), and amplifications were carried out using a C1000 Touch thermocyler (BioRad). Reactions were analyzed using a QX100 droplet reader (Bio-Rad) and quantified using QuantaSoft v.1.6.6 (Bio-Rad). Results were converted to copy number/g tissue extracted by accounting for sample preparation and dilution. To analyze the samples collected in 2015 a probe targeting strawberry *Rubisco* gene was developed (Supplementary Table S2), in order to express the results in terms of fractional abundance. Due to the lack of internal control assays, the results obtained from strawberry samples from 2014 along with the blueberry, blackberry, raspberry, periwinkle samples were converted to copy number/g tissue.

### Detection of SbGP/MPV *cpn60* using LAMP

Amplification primers (Supplementary Table S2) for LAMP were designed using LAMP Designer v. 1.12 (Premier Biosoft, Palo Alto, CA). LAMP conditions were as described for calcein detection^43^, and a LAMP temperature of 63 °C (1 hour assay time) was used. Alternatively, the same LAMP primers were used with detection using Isothermal Detection Reagent (Prolab Diagnostics, Richmond Hill, ON, Canada) at a temperature of 63 °C for 30 minutes. After amplification, an annealing curve was generated (90 °C–75 °C at 0.05 °C/sec). Reactions were monitored in real time using a Genie II or Genie III instrument (OptiGene, Horsham, UK), and the time to positive (T_p_) was reported by the instrument. For binomial (positive/negative) detection, reactions using calcein-based detection were viewed under ultraviolet light using a transilluminator (Bio-Rad Gel Doc). The 2015 samples were analyzed undiluted and diluted 1:50 with 10 mM Tris-Cl, pH 8.5 to examine the effect of co-purifying inhibitors.

### Determination of SbGP/MPV-targted assay parameters

The performance characteristics of the molecular diagnostic assays were determined according to established standards^55, 58^. Analytical specificity was examined by using as template for the LAMP and qPCR assays genomic DNA isolated from plants infected with the various phytoplasma strains described in Dumonceaux *et al*.^12^. Analytical specificity was also determined using as template a mixture of 10^6^ copies each of the cloned *cpn60* UT amplicons described in Dumonceaux *et al*.^12^. The linearity of the quantitative assays was examined by polynomial regression analysis of the C_q_ or T_p_ determined over a range of dilutions in a background of uninfected strawberry DNA. Detection limits were inferred by examining the lower levels of detection observed in these assays. Intra-assay precision was determined by performing up to 10 replicates and determining the standard deviation of the calculated results determined for four naturally infected samples. Finally, the performances of the direct PCR-based assays (*cpn60* and F2nR2) were compared by scoring the numbers of positive and negative results obtained using the two methods and calculating sensitivity and specificity as described^55^.

### SbGP/MPV phytoplasma distribution in central Mexico

Geospatial data for Mexico was downloaded from the GADM database of Global Administrative Areas (www.gadm.org) and plotted in R (http://www.R-project.org/) using ggplot2^59^. To assess the geographic distribution of SbGP/MPV phytoplasma, the qRT-PCR-determined SbGP/MPV genome copy number/g tissue was expressed on the map generated per locality and state.

## Electronic supplementary material

supplementary information
