# Supplementary material for: Molecular diagnostic assays based on cpn60 UT sequences reveal the geographic distribution of subgroup 16SrXIII-(A/I)I phytoplasma in Mexico
Source: Sci Rep. 2017 Apr 19;7:950. doi: 10.1038/s41598-017-00895-1 (PMC5430490; doi:10.1038/s41598-017-00895-1)
Supplement: Supplementary file 1 — supplementary information [file 41598_2017_895_MOESM1_ESM.pdf]

SCIENTIFIC REPORTS

Supplementary information

Molecular diagnostic assays based on *cpn60* UT sequences reveal the geographic distribution of subgroup 16SrXIII-(A/I)I phytoplasma in Mexico

Edel Pérez-López[a †], Douglas Rodríguez-Martínez[b], Chrystel Y. Olivier[c], Mauricio Luna-Rodríguez[d] and Tim J. Dumonceaux[c,e,*]

[a]Instituto de Biotecnología y Ecología Aplicada (INBIOTECA), Universidad Veracruzana, Avenida de Las Culturas Veracruzanas, Xalapa, Veracruz, México.

[b] Departamento de Investigación Aplicada, Driscoll´s, Zapopan, Jalisco, México.

[c]Agriculture and Agri-Food Canada, Saskatoon Research Centre, Saskatoon, Saskatchewan, Canada.

[d]Laboratorio de Alta Tecnología de Xalapa, Universidad Veracruzana, Xalapa, Veracruz, México.

[e]Department of Veterinary Microbiology, University of Saskatchewan, Saskatoon, Saskatchewan, Canada.

[†]Current address: Department of Entomology and Plant Pathology, Auburn University, Auburn, AL 36849, USA

*Corresponding author. **E-mail:** tim.dumonceaux@agr.gc.ca; **Tel:** 1-306-385-9450; **Fax:** 1-306-385-9482

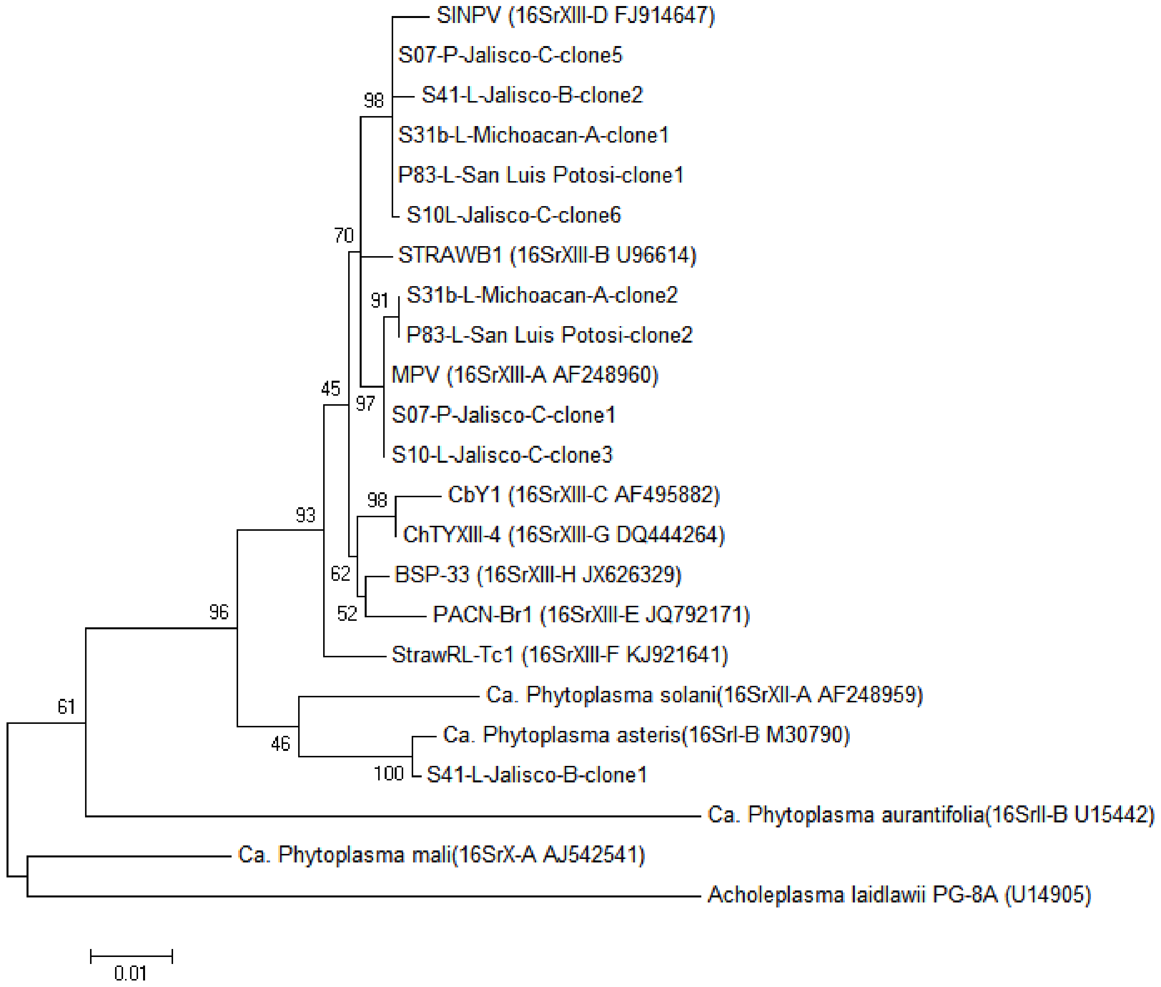

Figure S1. Phylogenetic tree reconstructed through the maximum likelihood method of the F2nR2 sequences obtained from samples S07-P-JC, S41-L-JB, and S10-L-JC, along with previously characterized phytoplasma strains. Accession numbers of the F2nR2 sequences obtained for the samples mentioned above are in the main text. Phylogenetic analysis was conducted using the maximum likelihood method with MEGA v6.0[1] and bootstrapping 1000 times to estimate stability. *Acholeplasma laidlawii* strain PG-8 A (U14905) was used as outgroup to root the tree. Bar, 1 substitution in 100 positions.

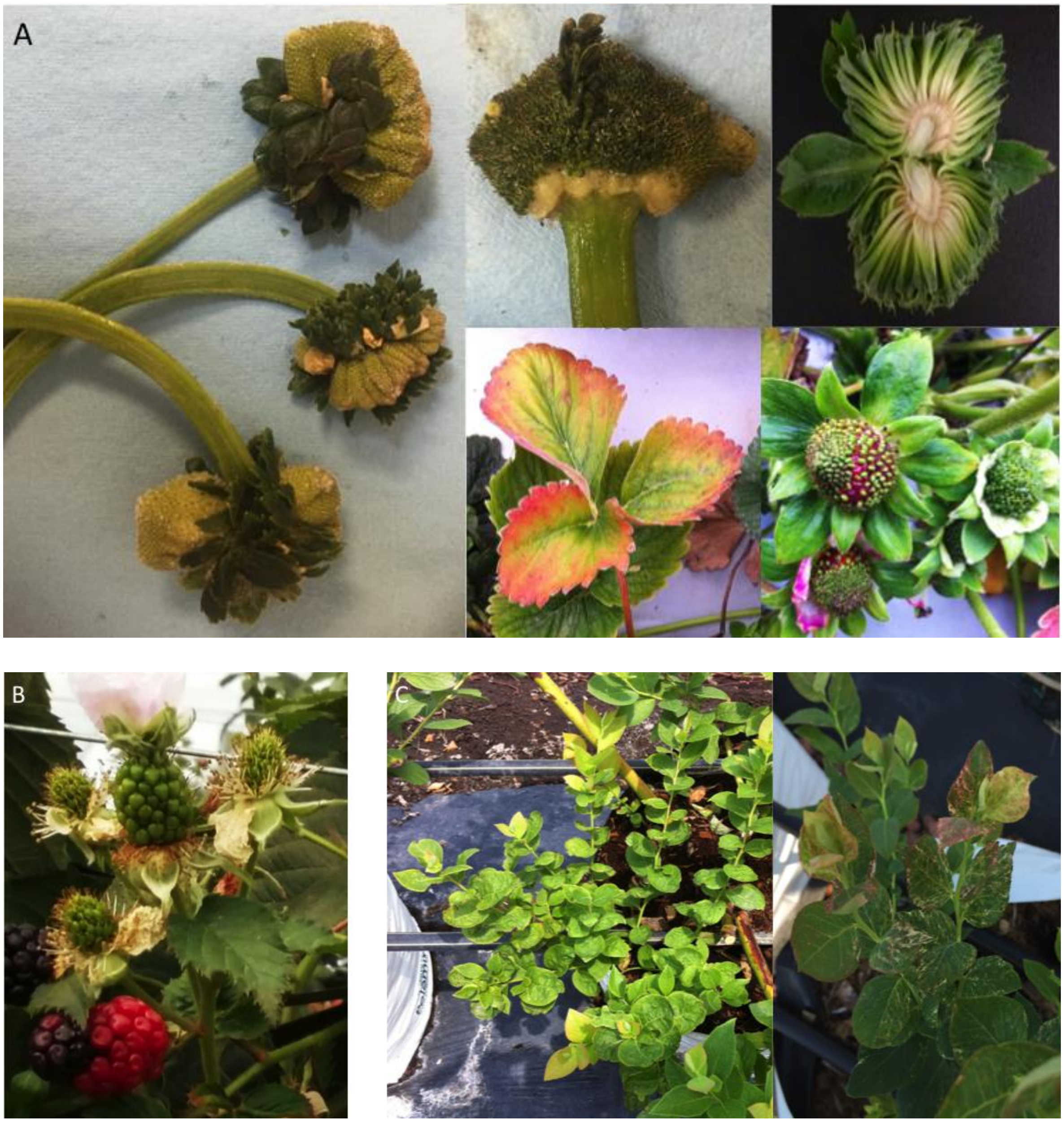
A
B
C

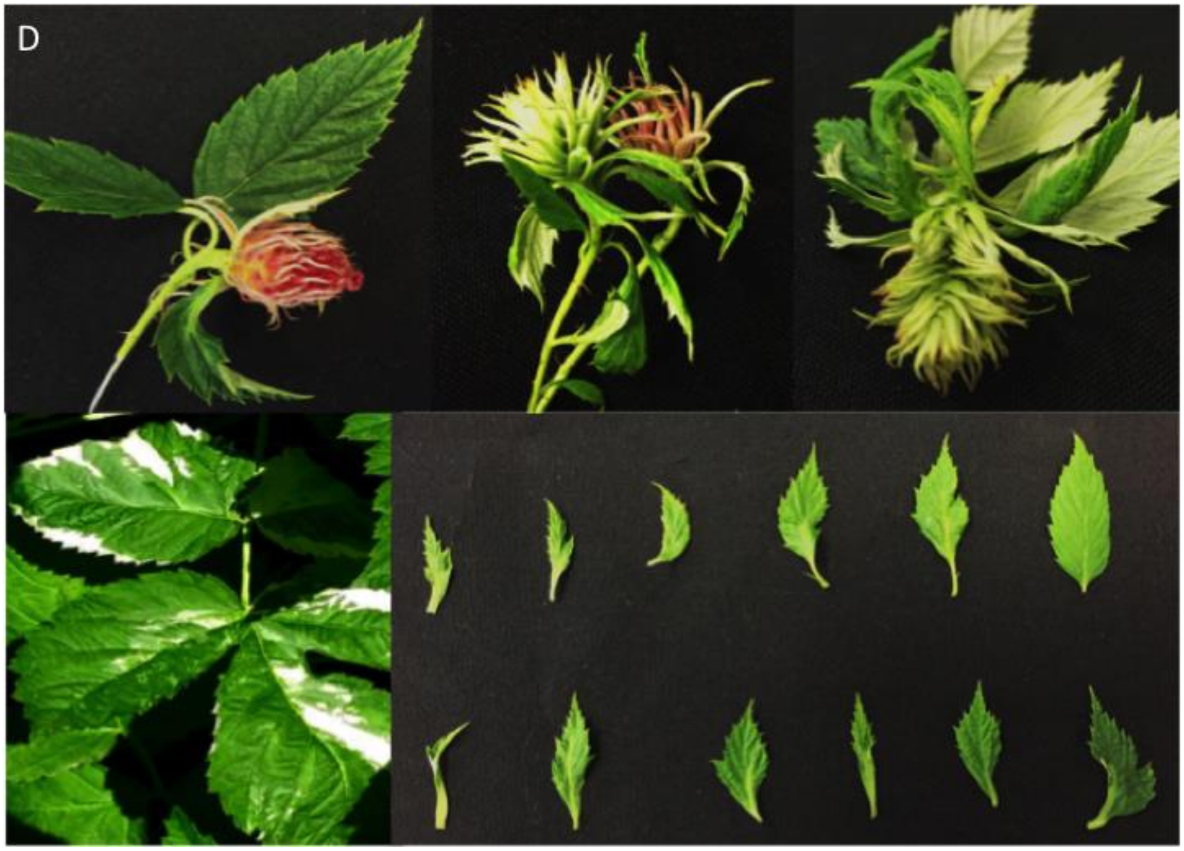

Figure S2. Symptoms detected in the berry plants analyzed in this study. (**A**) Strawberry plants showing symptoms previously described for SbGP disease featuring inedible fruit associated with the presence of green leaf-like structures. The symptoms depicted were associated with samples S12-P-JC, S10-P-JC, and S31-L-MB. (**B**) Blackberry showing inedible, yellowing fruit associated with the precense of green leaf-like structures (sample Bk01-L-JC). (**C**) Blueberry plants showing small leaves along with yellowing and redness in leaves (samples Bl01-L-JA and Bl02-L-JB). (**D**) Raspberry showing white leaf mosaic (R06-L-MC), little leaves and green structures in the fruits, a symptom referred to as ‘fruit witch´s broom’ (R01to 03-L-MA).

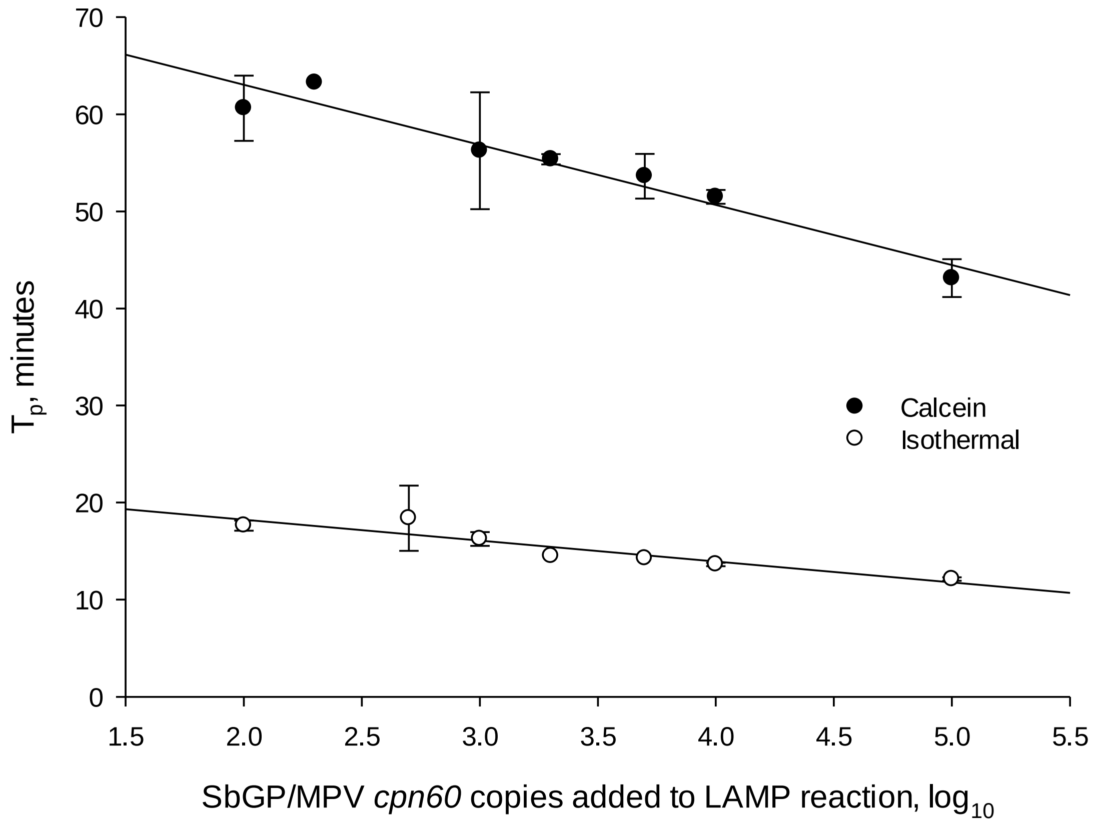

Fig. S3. LAMP assay linearity and detection limits. Assays were performed using calcein (closed circles) or isothermal (open circles) detection chemistry with known copy numbers of SbGP/MPV *cpn60* plasmids in a background of uninfected strawberry DNA.

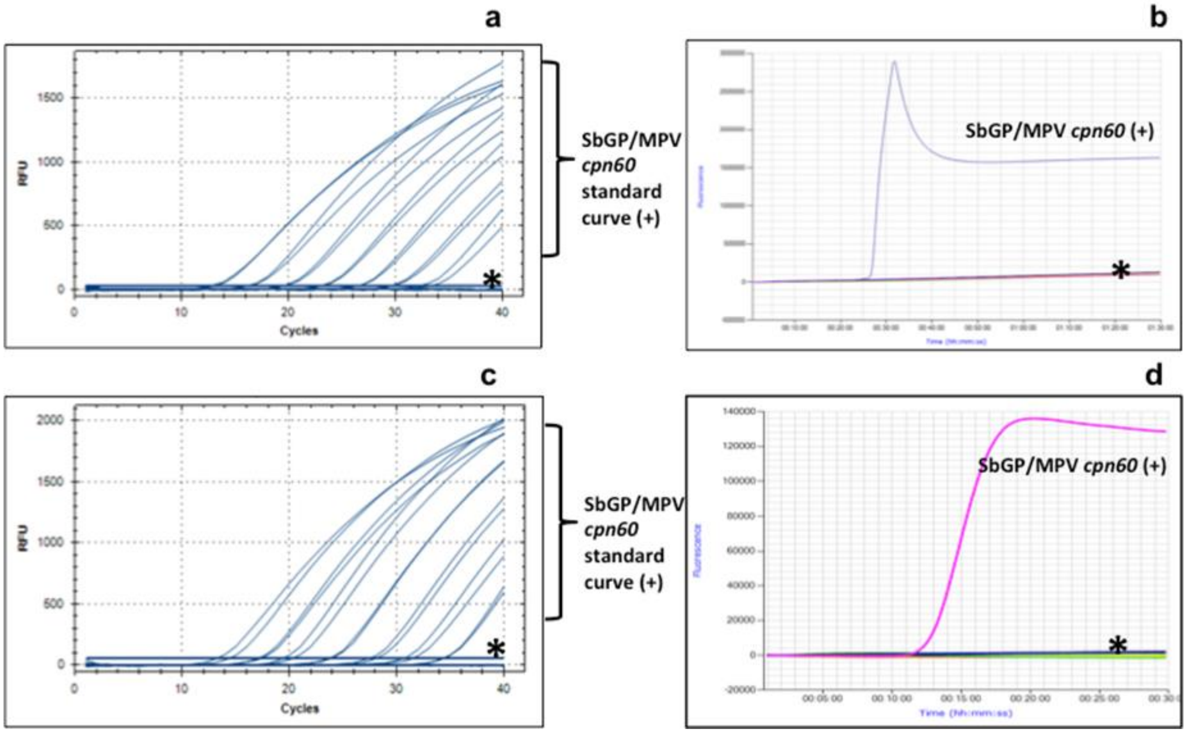

Fig. S4. Analytical specificity of the assays. **a.** and **c.** Quantitative PCR assay. **b.** and **d.** LAMP assay. **a.** and **b.** show a lack of assay signal (*) using no template as well as genomic DNA templates isolated from plants affected by the following strains: **AY-Ruta** (16SrI-A, ‘*Ca*. P. asteris- related strain), **SF1** (16SrI-B, ‘*Ca*. P. asteris’-related strain), **CVB, AY-Col** (16SrI-C, ‘*Ca*. P. asteris’-related strain), **RS** (16SrV-A, ‘*Ca*. P. ulmi’-related strain), **AshY** (16SrVII-A, ‘*Ca*. P. fraxini’-related strain), **Cr** (16SrIX-H, ‘*Ca*. P. phoenicium’-related strain), **AP** (16SrX-A, ‘*Ca*. P. mali’-related strain), **PYLR** (16SrX-C, ‘*Ca*. P. pyri’-related strain), **ESFY** (16SrX-F, ‘*Ca*. P. prunorum’-related strain), and **BN44948** (16SrXII-A, ‘*Ca*. P. solani’-related strain). **c.** and **d.** show a lack of signal (*) using no template control and using genomic DNA extracted from uninfected strawberry, blueberry, blackberry, raspberry, and periwinkle plants.

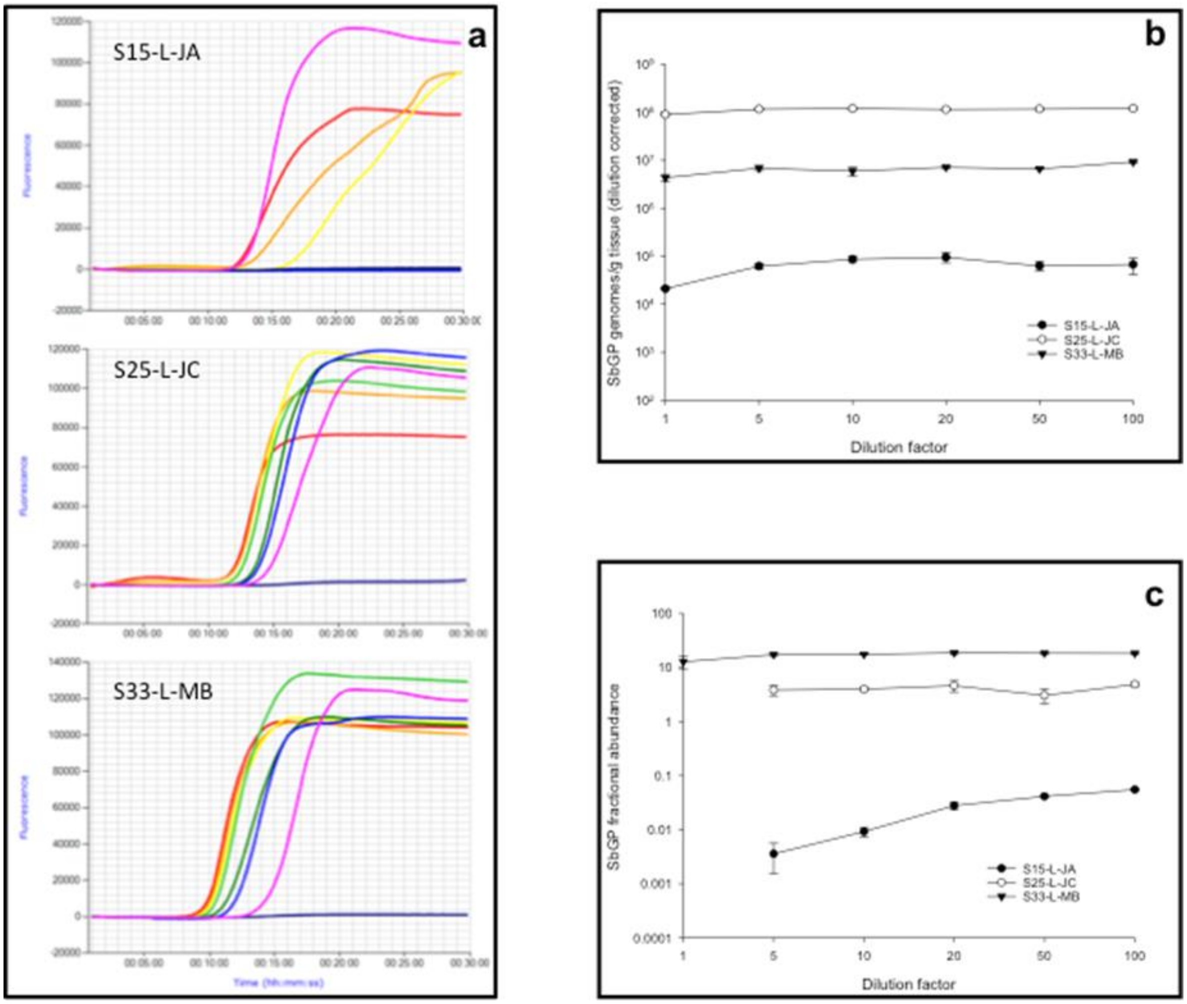

Fig. S5. Effect of dilution on the sensitivity of the LAMP, qRT- PCR, and ddPCR assays. **a.** Amplification curves obtained using LAMP assays where red is undiluted, orange 1:5, yellow 1:10, light green 1:20, dark green 1:50, light blue 1:10, dark blue no template control, and pink SbGP/MPV *cpn60* (+ control) of the corresponding sample. **b.** qRT-PCR dilution effect on the calculated number of genomes/g tissue. **c.** ddPCR dilution effect on the proportional abundance of SbGP genomes normalized to strawberry rubisco genes.

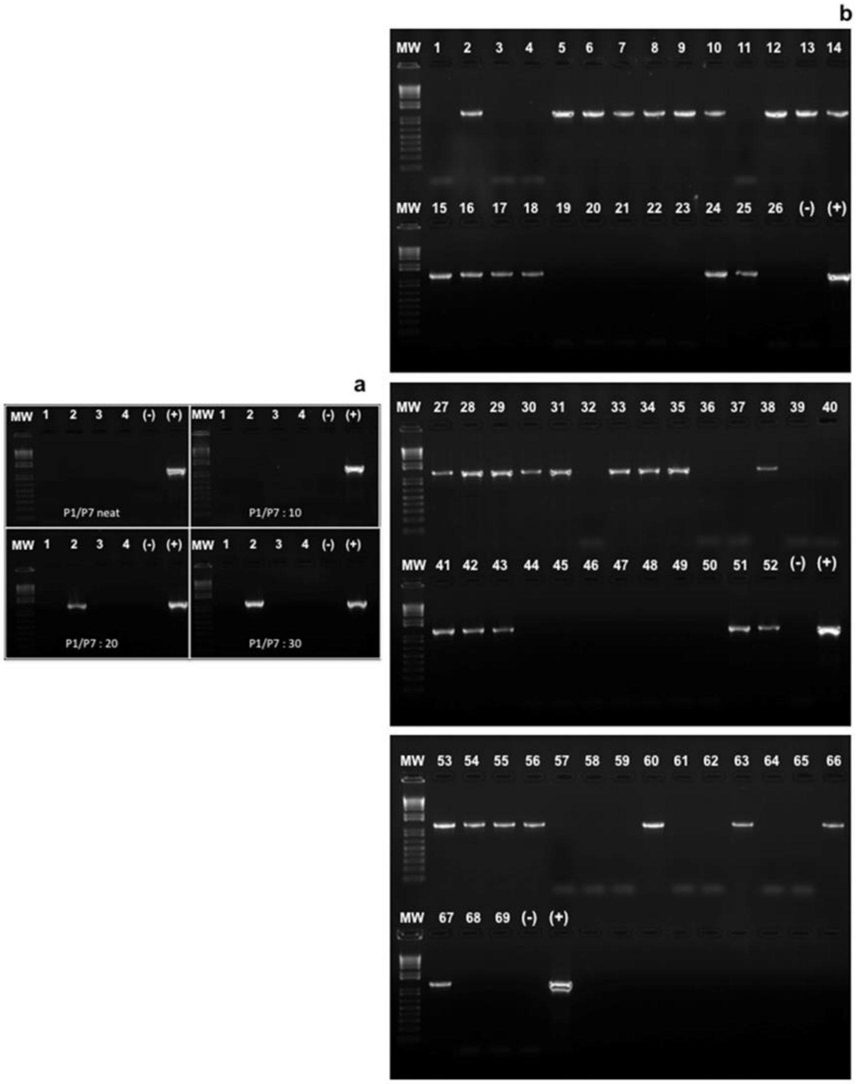

Fig. S6. F2nR2 gene fragment amplification of the samples collected in 2015 through nested PCR with P1/P7 primers in the first reaction, and R16F2n/R16R2 in the nested reaction. **a.** Dilution optimization of P1/P7 PCR product for use in the subsequent nested reaction. **b.** Electrophoresis of the nested PCR products of samples collected in 2015 with P1/P7 PCR product diluted 1:30. 1 to 55 is S01-L-MA to S43-L-JB (Table 1), 56 to 63 is R01-L-MA to R08-L-MC (Table 1), 64 to 65 is Bl01-L-JA to Bl02-L-JB (Table 1), 66 to 69 is Bk01-L-JC to Bk04-L-ME (Table 1).

Table S1. F2nR2 direct and nested and *cpn*60 direct PCR discordant samples confirmed by sequencing of *cpn*60 UT PCR product or the *cpn*60 UT-based molecular diagnostics developed in this study.

Sample	F2nR2 PCR	*cpn60* PCR	*cpn60* LAMP	Fluorescent microsphere hybridization	*cpn60* sequencing	*cpn60* qRT-PCR[a]	*cpn60* ddPCR[b]
S11-L-JC	+	+	+	SbGP/MPV	NT[c]	$3.86x10^7$	0.5650 (fractional abundance)
S33-L-MB	+	+	+	-	SbGP/MPV 100%	$2.02x10^6$	19.35 (fractional abundance)
S43-L-JB	+	+	+	-	ND[d]	$1.51x10^5$	0.0325 (fractional abundance)
R01-L-MA	+	+	+	NT	SbGP/MPV 100%	$1.87x10^5$	$2.66x10^6$ (genomes/g)
R01-P-MA	+	+	+	NT	NT	DNQ	$9.75x10^5$ (genomes/g)
R02-L-MA	-	+	+	NT	NT	DNQ	$1.31x10^5$ (genomes/g)
R03-L-MA	-	+	+	NT	ND	DNQ	$1.31x10^5$ (genomes/g)
R06-L-MC	-	+	+	NT	SbGP/MPV 100%	$1.66x10^5$	$3.32x10^6$ (genomes/g)
R08-L-MC	+	+	+	NT	ND	$1.03x10^5$	$3.15x10^6$ (genomes/g)
Bk01-L-JC	-	+	+	NT	ND	$8.87x10^4$	$3.13x10^6$ (genomes/g)
Bk03-L-MD	-	+	+	NT	SbGP/MPV 100%	$3.49x10^4$	$9.00x10^5$ (genomes/g)
Bl01-L-JA	+	+	+	NT	SbGP/MPV 100%	$1.34x10^5$	$2.79x10^6$ (genomes/g)
S22-L-MB	+	-	+	SbGP/MPV	ND	$1.40x10^6$	0.008 (fractional abundance)
S06-P-MB	+	-	+	SbGP/MPV	NT	$1.89x10^7$	1.24 (fractional abundance)

[a]qRT-PCR data expressed as genomes/g tissue
[b]ddPCR data expressed as fractional abundance for S (Strawberry) samples or genomes/g tissue for R (raspberry), Bk (blackberry), and Bl (blueberry) samples.
[c] NT, not tested.
[d]ND, not determined. Samples from raspberry, blueberry, and blackberry yielded low amounts of PCR product that provided poor quality sequence in many cases.

GenBank accession number of the *cpn60* UT sequenced from the samples: S33-L-MB (KY061181), R01-L-MA (KY061169), R06-L-MC (KY061172), Bk03-L-MD (KY061185), and Bl01-L-JA (KY061168).

Primer name	Sequence (5'-3')			Amplification conditions	
		Product size (bp)	Detection assay	qRT-PCR	ddPCR
D0414	AGGGGCTAATCCTATTTTC	132	qRT-PCR and ddPCR	1x 95°C, 3 min; 40 x 95°C, 10 sec; 56°C, 10 sec; 72°C, 30 sec (data collection)	1x 94°C, 10 min; 50x 94°C, 30 sec; 52°C, 1:00 (ramp 2°C/sec); 1x 98°C, 10 min
D0415	CCTGAAGAAATAGCTGCTA				
SbGP	FAM-ATTAGCGTC/ZEN/TCAAGTGGTTTCTCAAA-IB				
D0490	ACTACCTTGGCTCATTAT	92	ddPCR		
D0491	CCATGATTCTTCTGTCTATC				
Frag-rubisco[a]	HEX-CAACGGTCT/ZEN/ACTTCTTCACATC-IB				
MS31_2-F3	AAAGGATTTGAAACCGAGTTA	204	LAMP	63°C 60 minutes (calcein detection) or 30 minutes (isothermal detection)	
MS31_2-B3	CCCTTAATTTATTAGCAACCAA				
MS31_2-FIP	ATTCAACCGCCATAGATTCTCT-GAAGTAGTTCAAGGTTTACAAT				
MS31_2-BIP	AACCATTAGTTTTAGTGACCGA-TTCAACTGATTCAGCTACAATT				
MS31_2-loopF	AATACGGAGAAGCATATCCTT				
MS31_2-loopB	AGAAGGTGTTGTTAAAGAATCT				
SbGP[b]	ACCACTTGAGACGCTAAT	604	Fluorescent microsphere hybridization		

[a]Primers and probe designed using *Fragaria* x ananassa ribulose-bisphosphate carboxylase large subunit (*rbcL*), GenBank FAU06805
[b]hybridization capture probe used in combination with *cpn60*-targeted universal phytoplasma amplification primers as described [2]

Table S2. Sequences of amplification primers and hydrolysis probes used for detection and quantification of SbGP/MPV phytoplasmas.

Sample	DNA conc. ng/µl	PCR1 F2nR2 (1:50)	PCR2 *cpn60* -univ[a] (1:50)	PCR3 *cpn60*-FMA[b] (1:50)	FMA type	ddPCR SbGP[c]	ddPCR Sb-rubisco	qRT-PCR SbGP (genomes/ g tissue)	host	LAMP (calcein) neat	LAMP (calcein) 1:50	LAMP Time to positive (Tp), minutes Calcein 1:50	LAMP Time to positive (Tp), minutes Isothermal detection 1:50	Tanneal, °C
S26b-GP-MA	NT[d]	pos	pos	NT[d]	NT	$3.85X10^{8}$	NT	$1.12X10^{9}$	strawberry	pos	NT	35.38	NT	
S26b-P-MA	NT	NT[e]	NT	NT	NT	$4.23X10^{8}$	NT	$1.04X10^{9}$	strawberry	pos	NT	25.25	NT	
S26b-F-MA	NT	NT[e]	NT	NT	NT	$1.46X10^{8}$	NT	$3.98X10^{8}$	strawberry	pos	NT	38.38	NT	
S27b-GP-MA	NT	pos	pos	NT	NT	$6.92X10^{8}$	NT	$4.01X10^{9}$	strawberry	pos	NT	30.88	NT	
S31b-L-MA	NT	pos	pos	NT	NT	$2.41X10^{8}$	NT	$4.82X10^{8}$	strawberry	pos	NT	45.88	NT	
S31b-GP-MA	NT	NT[e]	pos	NT	NT	$4.80X10^{8}$	NT	$2.74X10^{9}$	strawberry	pos	NT	31.88	NT	
S31b-P-MA	NT	NT[e]	NT	NT	NT	$2.11X10^{8}$	NT	$4.34X10^{8}$	strawberry	pos	NT	35.00	NT	
S31b-F-MA	NT	NT[e]	NT	NT	NT	$5.48X10^{7}$	NT	$9.69X10^{7}$	strawberry	pos	NT	45.00	NT	
S267-GP-MA	NT	NT[e]	neg	NT	NT	neg	NT	$8.21X10^{2}$	strawberry	neg	NT	ND[f]	NT	
S267-P-MA	NT	NT[e]	NT	NT	NT	neg	NT	neg	strawberry	neg	NT	ND	NT	
S267-F-MA	NT	NT[e]	NT	NT	NT	neg	NT	neg	strawberry	neg	NT	ND	NT	
S289-L-MA	NT	pos	NT	NT	NT	$7.94X10^{7}$	NT	$1.34X10^{8}$	strawberry	pos	NT	49.63	NT	
S289-GP-MA	NT	NT[e]	NT	NT	NT	neg	NT	neg	strawberry	neg	NT	ND	NT	
S289-P-MA	NT	NT[e]	pos	NT	NT	$7.16X10^{7}$	NT	$4.24X10^{8}$	strawberry	pos	NT	35.38	NT	
S289-F-MA	NT	NT[e]	NT	NT	NT	neg	NT	neg	strawberry	neg	NT	ND	NT	
S01-L-MA	16.9	neg	neg	neg		0.0025	pos	DNQ[g]	strawberry	neg	neg	NT	neg	
S01-P-MA	9.72	pos	pos	pos	SbGP	2.6300	pos	$1.94X10^{7}$	strawberry	pos	pos	NT	13.5	81.14
S02-P-JA	6.32	neg	neg	neg		0.0095	pos	DNQ	strawberry	neg	neg	NT	neg	
S03-P-JB	5.28	neg	neg	neg		0.0000	pos	DNQ	strawberry	neg	neg	NT	neg	
S04-L-MB	14.4	pos	pos	pos	SbGP	3.5900	pos	$5.50X10^{7}$	strawberry	pos	pos	NT	15.25	81.09
S05-L-MB	8.08	pos	pos	pos	SbGP	2.7800	pos	$2.21X10^{7}$	strawberry	pos	pos	NT	13.75	81.09
S05-P-MB	56.8	pos	pos	pos	SbGP	5.5300	pos	$1.47X10^{8}$	strawberry	pos	pos	NT	13.75	81.14
S06-L-MB	38.2	pos	pos	pos	SbGP	6.5950	pos	$2.23X10^{8}$	strawberry	pos	pos	NT	13.5	81.14
S06-P-MB	37.7	pos	neg	pos	SbGP	1.2400	pos	$1.89X10^{7}$	strawberry	pos	pos	NT	13.75	81.09
S07-P-JC	14.1	pos	pos	pos	SbGP	16.0850	pos	$2.61X10^{8}$	strawberry	pos	pos	NT	12.5	81.24
S08-L-JA	13.4	neg	neg	neg		0.0200	pos	$2.09X10^{5}$	strawberry	neg	neg	NT	neg	
S09-L-MB	10.6	pos	pos	pos	SbGP	3.0100	pos	$1.83X10^{7}$	strawberry	pos	pos	NT	13	81.27
S09-P-MB	25.3	pos	pos	pos	SbGP	1.9150	pos	$5.04X10^{7}$	strawberry	pos	pos	NT	13.75	81.09

S10-L-JC	11.5	pos	pos	pos	SbGP	11.6700	pos	3.75X10^{8}	strawberry	pos	pos	NT		12.5	81.14
S10-P-JC	12.7	pos	pos	pos	SbGP	17.9500	pos	2.57X10^{8}	strawberry	pos	pos	NT		13.5	81.19
S11-L-JC	20.7	pos	pos	pos	SbGP	0.5650	pos	3.86X10^{7}	strawberry	pos	pos	NT		14	81.09
S12-P-JC	7.2	pos	pos	pos	SbGP	1.345	pos	6.52X10^{6}	strawberry	pos	pos	NT		15	81.14
S13-L-JC	11.4	pos	pos	pos	SbGP	2.905	pos	2.35X10^{7}	strawberry	pos	pos	NT		17.75	81.02
S14-P-JA	17	neg	neg	neg		0.013	pos	2.37X10^{5}	strawberry	pos	neg	NT	neg		
S15-L-JA	21.5	neg	neg	neg		0.0025	pos	6.26X10^{4}	strawberry	pos	pos	NT	neg		
S16-P-JA	9.88	neg	neg	neg		0.019	pos	DNQ	strawberry	pos	neg	NT	neg		
S17-P-JA	11	neg	neg	neg		0.007	pos	6.34X10^{4}	strawberry	pos	neg	NT	neg		
S18-P-JA	10.6	neg	neg	neg		0.0375	pos	2.54X10^{5}	strawberry	pos	neg	NT		22.75	81.09
S19-L-JD	8.48	pos	pos	pos	SbGP	3.305	pos	7.41X10^{7}	strawberry	pos	pos	NT		14.25	81.05
S19-P-JD	7	pos	pos	pos	SbGP	2.415	pos	2.16X10^{7}	strawberry	pos	pos	NT		14	81.15
S20-L-JD	12.8	neg	neg	neg		0.0025	pos	DNQ	strawberry	neg	neg	NT	neg		
S21-L-MB	8.56	pos	pos	pos	SbGP	0.64	pos	1.12X10^{7}	strawberry	pos	pos	NT		16.75	81.14
S21-P-MB	7.44	pos	pos	pos	SbGP	0.315	pos	1.60X10^{7}	strawberry	pos	pos	NT		15.5	81.15
S22-L-MB	14.2	pos	neg	pos	SbGP	0.008	pos	1.40X10^{6}	strawberry	pos	pos	NT		15.25	81.1
S22-P-MB	13.2	pos	pos	pos	SbGP	0.995	pos	1.07X10^{8}	strawberry	pos	pos	NT		13.5	81.06
S23-L-MB	15.4	pos	pos	pos	SbGP	14.195	pos	2.62X10^{8}	strawberry	pos	pos	NT		12.75	81.05
S24-P-JB	9.24	neg	neg	neg		0.0135	pos	DNQ	strawberry	pos	neg	NT	neg		
S25-L-JC	19.6	pos	pos	pos	SbGP	3.05	pos	8.51X10^{7}	strawberry	pos	pos	NT		14	81.07
S25-P-JC	3.97	pos	pos	pos	SbGP	1.365	pos	2.31X10^{8}	strawberry	pos	pos	NT		13.25	80.97
S26-L-JA	17.8	pos	pos	pos	SbGP	3.065	pos	8.38X10^{7}	strawberry	pos	pos	NT		14.25	80.95
S26-P-JA	15.2	neg	neg	neg		0.011	pos	4.64X10^{4}	strawberry	pos	pos	NT	neg		
S27-P-JA	0.896	neg	neg	neg		0	pos	0.00	strawberry	neg	neg	NT	neg		
S28-L-MB	0.808	pos	pos	pos	SbGP	6.95	pos	1.14X10^{6}	strawberry	pos	pos	NT		13.25	81.09
S29-L-MB	2.38	neg	neg	neg		0.16	pos	4.45X10^{4}	strawberry	pos	neg	NT	neg		
S30-P-MB	0.596	neg	neg	neg		0.07	pos	0.00	strawberry	pos	neg	NT	neg		
S31-L-MB	0.832	pos	pos	neg		2.3	pos	3.02X10^{5}	strawberry	pos	pos	NT		12.5	81.04
S32-L-MB	0.952	pos	pos	neg		2.55	pos	3.20X10^{5}	strawberry	pos	pos	NT		12	81
S33-L-MB	0.876	pos	pos	neg		19.35	pos	2.02X10^{6}	strawberry	pos	pos	NT		10.75	80.91
S34-L-MC	1.21	neg	neg	neg		0	pos	2.18X10^{5}	strawberry	pos	neg	NT		15.25	80.96
S35-L-MD	1.44	neg	neg	neg		0.055	pos	DNQ	strawberry	pos	pos	NT		10.25	80.78
S36-L-ME	5.56	neg	neg	neg		5.95	pos	0.00	strawberry	neg	pos	NT		11.5	80.81
S37-P-MD	1.2	neg	neg	neg		0	pos	DNQ	strawberry	pos	neg	NT		15.5	81.3

S38-L-MF	2.66	neg	neg	neg		0.04	pos	DNQ	strawberry	pos	pos	NT		12.25	80.8
S39-L-MG	2.03	neg	neg	neg		NT	pos	0.00	strawberry	neg	neg	NT		12.5	81.16
S40-L-MH	1.29	neg	neg	neg		0.04	pos	0.00	strawberry	neg	neg	NT		12.25	80.81
S41-L-JB	8.4	pos	pos	pos	AY-OY-M	NT	pos	$6.52X10^{4}$	strawberry	pos	pos	NT		10.5	80.92
S41-P-JB	1.8	pos	pos	pos	AY-OY-M	0.0565	pos	$5.02X10^{4}$	strawberry	pos	pos	NT		11	80.92
S42-L-JB	3.43	pos	pos	neg		0.605	pos	$1.64X10^{6}$	strawberry	pos	pos	NT		9.75	80.89
S43-L-JB	6.16	pos	pos	neg		0.0325	pos	$1.51X10^{5}$	strawberry	pos	pos	NT		10	80.92
R01-L-MA	5.6	pos	pos	NT		$2.66X10^{6}$	NT	$1.87X10^{5}$	raspberry	pos	pos	NT		9.75	81.05
R01-P-MA	1.44	pos	pos	NT		$9.75X10^{5}$	NT	DNQ	raspberry	pos	pos	NT		10.5	81.15
R02-L-MA	12.9	neg	neg	NT		$1.31X10^{5}$	NT	DNQ	raspberry	pos	pos	NT		9.25	81.07
R03-L-MA	3.7	neg	pos	NT		$1.31X10^{5}$	NT	DNQ	raspberry	pos	pos	NT		9.5	81.02
R04-L-MB	7.96	neg	neg	NT		$1.11X10^{6}$	NT	$5.36X10^{4}$	raspberry	pos	pos	NT		9.25	81
R05-L-MC	17.1	pos	pos	NT		$5.44X10^{6}$	NT	$2.05X10^{5}$	raspberry	pos	pos	NT		9.5	81
R06-L-MC	4.2	neg	pos	NT		$3.32X10^{6}$	NT	$1.66X10^{5}$	raspberry	pos	pos	NT		9.5	81.05
R07-L-MC	9	neg	neg	NT		$7.13X10^{5}$	NT	DNQ	raspberry	pos	pos	NT		9.5	81.2
R08-L-MC	4.92	pos	pos	NT		$3.15X10^{6}$	NT	$1.03X10^{5}$	raspberry	pos	pos	NT		11.75	82.1
Bk01-L-JC	1.74	neg	pos	NT		$3.13X10^{6}$	NT	$8.87X10^{4}$	blackberry	pos	pos	NT		11.5	80.86
Bk02-L-JD	3.54	pos	pos	NT		$1.50X10^{6}$	NT	$4.22X10^{4}$	blackberry	pos	pos	NT		9.5	80.99
Bk03-L-MD	19.9	neg	pos	NT		$9.00X10^{5}$	NT	$3.49X10^{4}$	blackberry	pos	pos	NT		8.75	81.09
Bk04-L-ME	26.5	neg	neg	NT		0.00	NT	DNQ	blackberry	neg	neg	NT	neg		
Bl01-L-JA	3.56	pos	pos	NT		$2.79X10^{6}$	NT	$1.34X10^{5}$	blueberry	pos	pos	NT		9	81.05
Bl02-L-JB	3.01	neg	neg	NT		0.00	NT	DNQ	blueberry	pos	pos	NT		10.25	81.11
P83-L-SLP		pos	pos	NT		$2.53X10^{9}$	NT	$4.67X10^{9}$	periwinkle	pos	NT	38.42	NT		
P86-L-SLP		pos	pos	NT		$2.82X10^{9}$	NT	$6.13X10^{9}$	periwinkle	pos	NT	37.08	NT		

[a]*cpn60*-univ, PCR using *cpn60*-targeted universal phytoplasma primers[2]

[b]FMA, fluorescent microsphere hybridization assay

[c]ddPCR data expressed as genomes/g tissue extracted where internal control data is NT; otherwise expressed as fractional abundance

[d]NT, Not tested

[e]sample was neg using 16S-targeted pan-phytoplasma primers P1-Tint[3]

[f]ND, Not detected

[g]DNQ, Detectable but not quantifiable

Table S3. Complete results of all assays on all samples.

Sample	qRT-PCR genomes/g tissue	
	Mean (n)	Standard deviation
S03-P-JB	DNQ (2)	-
S24-P-JB	DNQ (2)	-
S41-L-JB	$7.68x10^6$ (2)	$1.03x10^6$
S41-P-JB	$8.61x10^6$ (2)	$5.07x10^6$
S42-L-JB	0 (2)	0
S43-L-JB	DNQ (2)	-
Bl02-L-JB	DNQ (2)	-

Table S4. Results obtained using AY-specific qRT-PCR on samples obtained from Jalisco-location B.

REFERENCES

1 Tamura, K., Nei, M. & Kumar, S. Prospects for inferring very large phylogenies by using the neighbor-joining method. *Proc. Natl. Acad. Sci. U.S.A.* **101**, 11030-11035, doi:10.1073/pnas.0404206101 (2004).

2 Dumonceaux, T. J., Green, M., Hammond, C., Perez, E. & Olivier, C. Molecular diagnostic tools for detection and differentiation of Phytoplasmas based on chaperonin-60 reveal differences in host plant infection patterns. *PLoS ONE* **9**, e116039, doi:10.1371/journal.pone.0116039 (2014).

3 Smart, C. D. *et al.* Phytoplasma-specific PCR primers based on sequences of the 16S-23S rRNA spacer region. *Appl. Environ. Microbiol.* **62**, 2988-2993 (1996).
